# High-specificity bioinformatics framework for epigenomic profiling of discordant twins reveals specific and shared markers for ACPA and ACPA-positive rheumatoid arthritis

**DOI:** 10.1186/s13073-016-0374-0

**Published:** 2016-11-22

**Authors:** David Gomez-Cabrero, Malin Almgren, Louise K. Sjöholm, Aase H. Hensvold, Mikael V. Ringh, Rakel Tryggvadottir, Juha Kere, Annika Scheynius, Nathalie Acevedo, Lovisa Reinius, Margaret A. Taub, Carolina Montano, Martin J. Aryee, Jason I. Feinberg, Andrew P. Feinberg, Jesper Tegnér, Lars Klareskog, Anca I. Catrina, Tomas J. Ekström

**Affiliations:** 1 Center for Molecular Medicine at Karolinska Institutet and Karolinska University Hospital, Stockholm, Sweden; 2 Department of Medicine, Unit of Computational Medicine, Stockholm, Sweden; 3 Bioinformatic Infrastructure for Life Sciences, Stockholm, Sweden; 4 Department of Clinical Neuroscience, Karolinska Institutet, Stockholm, Sweden; 5 Department of Medicine, Unit of Rheumatology, Karolinska University Hospital Solna, Stockholm, Sweden; 6 Center for Biosciences, Department of Biosciences and Nutrition, Karolinska Institutet, Stockholm, Sweden; 7 Translational Immunology Unit, Department of Medicine Solna, Karolinska Institutet and Karolinska University Hospital, Stockholm, Sweden; 8 Center for Epigenetics, Johns Hopkins University, Baltimore, MD USA; 9 Department of Medicine, Johns Hopkins University, Baltimore, MD USA; 10 Departments of Biostatistics, Johns Hopkins University, Baltimore, MD USA; 11 Departments of Mental Health, Bloomberg School of Public Health, Johns Hopkins University, Baltimore, MD USA; 12 Medical Scientist Training Program, and Predoctoral Training Program in Human Genetics, McKusick-Nathans Institute of Genetic Medicine, Johns Hopkins University School of Medicine, Baltimore, MD USA; 13 Departments of Pathology, Massachusetts General Hospital, Charlestown, MA USA; 14 Harvard Medical School, Boston, MD USA; 15 Biostatistics, Harvard TH Chan School of Public Health, Boston, MA USA; 16 Broad Institute of Harvard and MIT, Cambridge, MA USA; 17 Mucosal and Salivary Biology Division, King’s College London Dental Institute, London, UK; 18 Department of Clinical Science and Education, Karolinska Institutet, and Sachs’ Children and Youth Hospital, Södersjukhuset, Stockholm, Sweden

**Keywords:** Rheumatoid arthritis, ACPA, DNA methylation, Epigenetics, Bioinformatics

## Abstract

**Background:**

Twin studies are powerful models to elucidate epigenetic modifications resulting from gene–environment interactions. Yet, commonly a limited number of clinical twin samples are available, leading to an underpowered situation afflicted with false positives and hampered by low sensitivity. We investigated genome-wide DNA methylation data from two small sets of monozygotic twins representing different phases during the progression of rheumatoid arthritis (RA) to find novel genes for further research.

**Methods:**

We implemented a robust statistical methodology aimed at investigating a small number of samples to identify differential methylation utilizing the comprehensive CHARM platform with whole blood cell DNA from two sets of twin pairs discordant either for ACPA (antibodies to citrullinated protein antigens)-positive RA versus ACPA-negative healthy or for ACPA-positive healthy (a pre-RA stage) versus ACPA-negative healthy. To deconvolute cell type-dependent differential methylation, we assayed the methylation patterns of sorted cells and used computational algorithms to resolve the relative contributions of different cell types and used them as covariates.

**Results:**

To identify methylation biomarkers, five healthy twin pairs discordant for ACPAs were profiled, revealing a single differentially methylated region (DMR). Seven twin pairs discordant for ACPA-positive RA revealed six significant DMRs. After deconvolution of cell type proportions, profiling of the healthy ACPA discordant twin-set revealed 17 genome-wide significant DMRs. When methylation profiles of ACPA-positive RA twin pairs were adjusted for cell type, the analysis disclosed one significant DMR, associated with the *EXOSC1* gene. Additionally, the results from our methodology suggest a temporal connection of the protocadherine beta-14 gene to ACPA-positivity with clinical RA.

**Conclusions:**

Our biostatistical methodology, optimized for a low-sample twin design, revealed non-genetically linked genes associated with two distinct phases of RA. Functional evidence is still lacking but the results reinforce further study of epigenetic modifications influencing the progression of RA. Our study design and methodology may prove generally useful in twin studies.

**Electronic supplementary material:**

The online version of this article (doi:10.1186/s13073-016-0374-0) contains supplementary material, which is available to authorized users.

## Background

Epigenetic states define the functional genome and its communication with, and response to, the environment [1]. Importantly, epigenetic modifications have been shown to be partly associated with the genetic and environmental backgrounds in which they reside [2, 3]. Since epigenetic mechanisms are believed to be important players in the interactions between genome and environment, it is essential to separate the genetic and epigenetic components in order to elucidate the mechanisms by which the environment may impact on the genome and phenotypes. This can be approached by studies of monozygotic twins with discordant phenotypes.

One of the basic epigenetic mechanisms is DNA methylation, which is closely associated with gene regulation both near and distant to genes. The pattern of DNA methylation of “CpG island shores”, 1–2 kb downstream or upstream of CpG islands, has been found to associate strongly with cell type as well as disease [4]. Furthermore, distant enhancer regions may also employ methylation in gene regulatory machineries [5].

Rheumatoid arthritis (RA) is a systemic inflammatory disease affecting approximately 1% of the human population [6] with a multifactorial etiology [7, 8]. RA development has been serologically investigated using retrospective collected samples [9, 10], and more recently prospective collected biobank samples from individuals at increased risk for RA [11–13] have allowed identification of several distinct phases of disease development [14]. One of these initial phases is characterized by signs of deregulated immune system function with the presence of disease-specific autoantibodies—referred to as anti citrullinated peptide antibodies (ACPA)—directed to tissue antigens expressed in the joints. These antibodies can be detected already a decade prior to clinical RA symptoms [9, 15]. ACPA-positive RA disease development is therefore thought to be an accessible developmental prototype of a complex autoimmune disease. The normal maintenance of the immune system, as well as the failure to regulate it, is dependent on epigenetic factors [16]. RA is a complex autoimmune disease in which epigenetic changes have been shown to mediate previously unrecognized genetic effects [2].

ACPA-positive RA is the major form of RA, its etiology involving genetic predisposition in combination with exposure to certain environmental risk factors [7, 8, 17, 18]. Twin, family, and genetic studies have shown that environmental factors make a substantial contribution, besides the genetic factors, to the development of ACPA and ACPA-positive RA [11, 19, 20]. More specifically, the risk for development of ACPA and ACPA-positive RA is associated with smoking and HLA-*DRB1* gene alleles [17, 18]. In addition, over 100 non-MHC risk alleles for ACPA-positive RA have been identified [21]. Our recent finding of associations between genotype, DNA methylation, and ACPA-positive RA within the *HLA* cluster [2] provide genetic insight into how epigenetic regulation can mediate early stages of the disease. Yet, little is known in general of how and if environmental factors orchestrate epigenetic changes before disease onset.

In our previous work, we showed how epigenetic changes in RA can mediate previously opaque genetic differences [2]. Here we wished to understand epigenetic changes where the genome has a homogenous background by employing monozygotic (MZ) twins. In order to understand some of the mechanistic changes in RA development, we set out to examine the DNA methylation profile in two MZ twin sets, discordant for two different phases of disease development, by using the “comprehensive high-throughput arrays for relative methylation” (CHARM) technology, which employs 2.1 million probes [22] grouped in 43,897 genomic regions. The CHARM array also includes 4500 control probes allowing unmethylated regions to be associated, on average, with values of 0 [22]. Coverage information for the CHARM array design is depicted in Additional file 1: Figure S1.

Since the sample sizes are usually small in twin studies interrogating discordant situations, a robust methodological framework was developed to identify changes in DNA methylation with high specificity, minimizing the number of false positives (low-sensitivity). Our paired data analysis suggests that the employed MZ twin model does indeed isolate epigenetic RA determinants from genetic ones, and also may identify candidate biomarkers associated with a temporal epigenetic trajectory of disease development. Importantly, by estimating the proportion of the common cell types in the peripheral blood samples, we were able to distinguish phenotype-driven epigenetic changes from cell type-driven ones. Our results reveal differentially methylated loci in the twin sets that discriminate ACPA-positive healthy subjects from those with ACPA-positive RA, some of which are replicated in a previously analyzed non-twin cohort, as well as also suggesting novel associated genes.

## Methods

### Clinical material

DNA was obtained from five healthy MZ twin pairs discordant for ACPA and seven MZ twin pairs discordant for ACPA-positive RA (Table 1; Additional file 2: Table S1). For the replication with bisulfite pyrosequencing (see the “Statistical analysis for validation” section in the “Methods”) an additional six healthy MZ twin pairs discordant for ACPA and six MZ twin pairs discordant for ACPA-positivity (Additional file 2: Table S1) were analyzed. The 24 twin pairs belong to a population-based twin cohort (Twingene) which is part of the Swedish Twin Registry [11, 23]. Information about smoking habits, C reactive protein, and occurrence of the HLA-*DRB1* shared epitope (SE) are listed in Table 1. ACPA presence was tested by CCP2 ELISA assay (Immunoscan CCPlus) using the cutoff set by the manufacturer to define positive sera [11]. Each individual gave written approval for participation in the study and the ethical review board at the Karolinska Institutet approved the study.

**Table 1:** Summary information of the individuals selected for the experimental design

Discordance type	Number of twin pairs	Females	Ever smokers	SE occurrence	Median age (years) at blood sampling (IQR)	Median CRP at blood sampling (IQR)
ACPA-positive healthy	5	80%	30%	60%	63 (62–74)	1.7 (1.2–4.2)
ACPA-positive RA	7	43%	36%	71%	70 (68–72)	3.7 (2.6–7.9)

*SE* shared epitope, *IQR* interquartile range, *CRP* C-reactive protein

### ACPA-positive healthy: verification and discordance status

ACPA-positive healthy discordant twins tested positive for ACPA (high concentration, >75 AU/ml) while their sibling tested negative for ACPA. None of the twins had self-reported chronic rheumatic joint disease at the time of blood collection. Also, none of these twins was identified with a discharge RA diagnosis (or other rheumatic joint disease diagnosis, e.g., polyarthritis) in the Swedish National Patient Register for a median time period of 3 years (interquartile range (IQR) 2–4) following blood collection.

### ACPA-positive RA: verification and discordance status

The ACPA-positive RA discordant twins tested positive for ACPA (high concentration, > 75 AU/ml) while their healthy siblings tested negative for ACPA. Also, these ACPA-positive twins had self-reported RA at the time of blood sampling. The self-reported RA diagnosis was verified by both linkage to the Swedish National Patient Register and review of the medical records according to the American College of Rheumatology 1987 criteria [24]. None of the ACPA-negative siblings had self-reported chronic rheumatic joint disease at the time of blood collection. Also, none of these ACPA-negative siblings had previously been discharged with a RA diagnosis (or other rheumatic joint disease diagnosis, e.g., polyarthritis) in the Swedish National Patient Register for a median time period of 3 years (IQR 2–4) following blood collection.

### Sampling and DNA extraction

The twins donated peripheral blood at outpatient clinics. Sera and tubes with whole blood were sent to Karolinska University Laboratory by overnight post and then forwarded to the KI Biobank. At the KI Biobank the DNA was extracted using the Puregene extraction kit (Gentra Systems, Minneapolis, MN, USA). After extraction the DNA was subsequently stored with a barcode at −20 °C. Quality control was done by 1% agarose gel to detect degradation. Sera was aliquoted and stored with a barcode in liquid nitrogen (−180 °C) at the KI Biobank.

### Low resolution typing HLA-*DRB*

Two-digit HLA*-DRB1* typing was conducted using sequence-specific primer PCR (DR low-resolution kit (2-digit); Olerup SSP, Saltsjöbaden, Sweden) and the PCR products were loaded on 2% agarose gels. To determine the specific genotype, an interpretation table was used according to the manufacturer’s instructions. HLA-*DRB1* SE alleles were defined as *01 (except *0103), *04, and *10.

### DNA preparation and CHARM

DNA (1 μg per sample) was sheared, McrBC-digested, and gel fractionated before labeling and hybridization onto arrays covering 2.1 million CpG sites according to the protocol in [25]. CHARM is a method developed to analyze genome-wide gene-specific methylation that combines a purpose-made array design and a statistical procedure. The CHARM statistics-based algorithm first involves the identification of consecutive differential methylation sites, identifying them as candidate (differentially methylated regions (DMRs)) and second uses a boot-strapping approach to compute a significance level for each DMR [22]. This protocol also covers CpGs in lower CpG density regions of the genome, in addition to CpG islands and shores, and employs a smoothing algorithm allowing correction for CpG density and fragment biases which may otherwise occur in methyl-enrichment or methyl-depletion DNA fractionation methods. DNA from peripheral blood cells (PBC) was analyzed to determine the locus-specific differential methylation patterns. The method is comprehensively described in [22] and briefly in Additional file 3: Methods. TS1 (Twin-set of ACPA-positive healthy discordant twins) and TS2 (Twin-set of ACPA-positive RA discordant twins) were profiled separately in two batches; within each batch, all samples (e.g., healthy controls and RA individuals) were profiled together. In our analysis, and similarly to epigenome-wide association studies [26], we note the occurrence of a *batch effect* between TS1 and TS2, probably due to handling or processing effects; hence, we do not compare TS1 and TS2 statistically. Note that all chromosomal locations are based on the hg18 build (original CHARM design). When annotation to genes was conducted we applied *liftOver* to map to hg19 in order to confirm that the DMR-gene annotation was consistent between genome reference versions. We observed minor differences between DMR-gene mapping in hg18 and hg19.

### Array pre-processing

In the processing of CHARM arrays, several quality controls are considered: (1) the signal of background probes; (2) the standard deviation of untreated channel signals, which must be small; (3) the difference between the medians of control (CpG-free regions) and non-control probes, which must be negative; and (4) probes with a probe quality lower than 80 were discarded (see CHARM Bioconductor package for details [27]). In addition, after normalization, a quality control is applied to ensure that high correlation between samples is observed. For a more comprehensive discussion, see Additional file 3: Methods. Because DNA methylation profiling and CHARM arrays were processed in two different batches (for TS1 and TS2 associated samples, respectively), the data preprocessing and following analytical steps were performed separately for each batch.

### Methylation estimation and normalization

The methp function of the CHARM Bioconductor package [27] was used to estimate methylation percentages from signal intensities. A three-step methodology was used: (1) within-sample normalization (using non-CpG probes as a reference for unmethylated DNA values); (2) between-sample normalization by subset quantile normalization; and (3) percentage methylation estimation [27]. We considered sub-quantile normalization and LOESS normalization for between- and within-sample normalization, respectively.

### Quality control for confounders

**Fig. 1:**
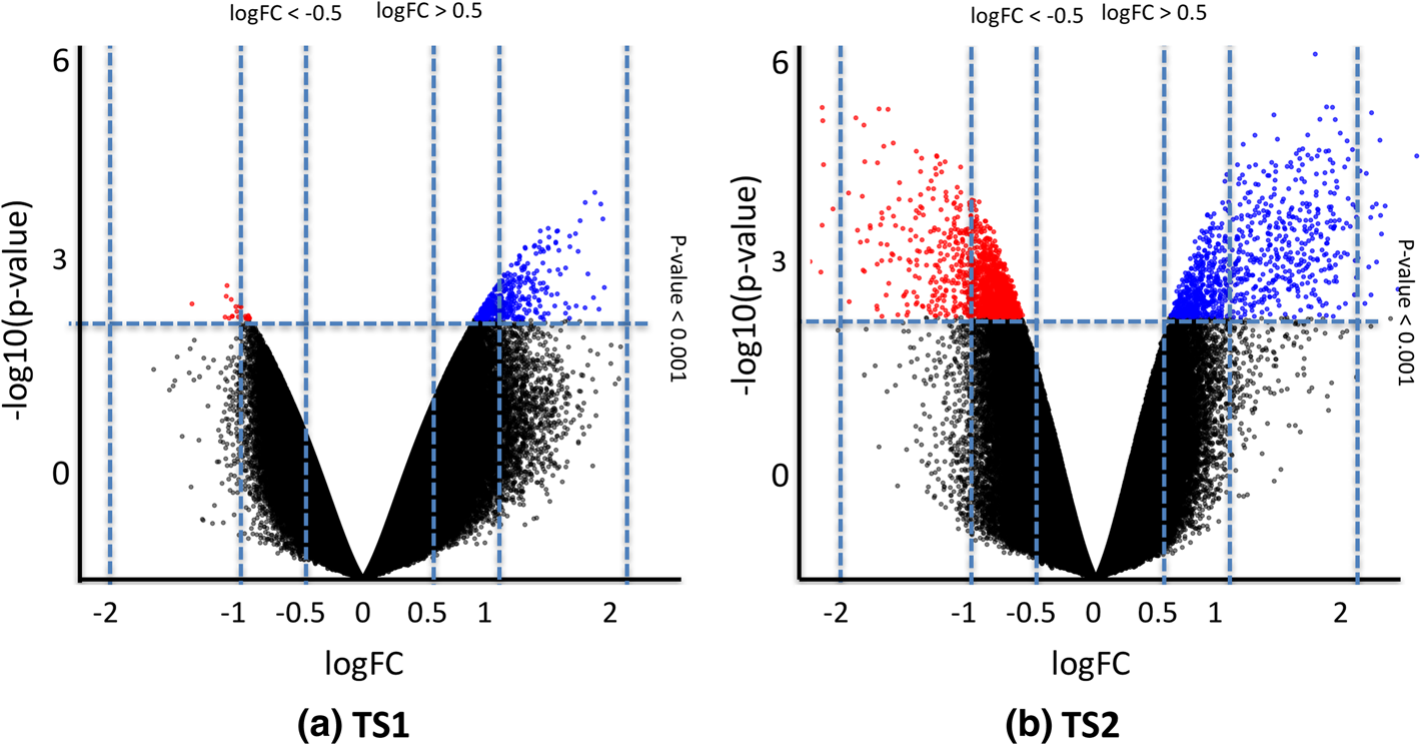
Differential methylation after correction for cell proportion. Log-transformed *p* value (*y-axis*) versus log-transformed fold change (*logFC*; *x-axis*) from the associated linear model. A negative logFC denotes hypomethylation and a positive logFC hypermethylation in TS1 and TS2 comparisons; the logFC was computed using M values in a linear model and using estimated cell proportions as covariates (see “Methods”). The *horizontal* and *vertical lines* are arbitrary thresholds selected to highlight possible tendencies. **a** Results from ACPA-positive healthy versus ACPA-negative healthy twin siblings (TS1). **b** Results from ACPA-positive RA versus ACPA-negative healthy twin siblings (TS2)

Possible confounders were investigated with regard to their association with experimental design (batch, within TS1 and TS2 separately) and clinical information (age, gender, and smoking). Also, the global variability of samples was investigated using multi-dimensional scaling (MDS; Additional file 1: Figure S26.); briefly, MDS depicts in two dimensions the associations between samples. We did not identify any associations for confounders using MDS, sva, or principal component analysis in TS1 samples. However, TS2 samples grouped by age and gender (Additional file 1: Figure S26b). To investigate the possible association of age and gender with the skewness observed in TS2 (Fig. 1b), we compared the distribution of differences in methylation within female pairs and within male pairs separately; no significant difference was found using a Kolmogorov–Smirnov test (*p* value >0.2) and the top differentially methylated probes. Similar results were obtained when considering age and dividing samples into two groups: “aged more than 70 years” and “aged less than 70 years” (*p* value >0.2).

### Single probe analysis

The same linear model used in dmrFind (CHARM Bioconductor package [27]) for DMR discovery was also used to compute *p* values for individual probes. No single probe was found to be differentially methylated when considering a false discovery rate (FDR) <0.20 using the Benjamini–Hochberg FDR controlling procedure [28].

### DMR candidate identification

The dmrFind algorithm is described in detail in [22] and further explanation is given in Additional file 3: Methods. When cell proportion information is considered, the percentages of neutrophils, natural killer (NK) cells (CD56^+^), and the sum of CD4^+^ and CD8^+^ T cells are included as covariates.

### Resampling-based family-wise error rate

The CHARM algorithm provides three different statistics that are computed for each candidate DMR: (a) *avg*, the average (across probes) percentage methylation difference; (b) *max*, the maximum percentage methylation difference; and (c) *area.raw*, the number of probes multiplied by the averaged difference of methylation, which is the default mode. By running the default mode we observed that the selection from the list of candidate DMRs was biased towards DMR with larger numbers of probes (Additional file 1: Figures S24 and S25). To correct for this bias, we estimated for each candidate DMR the family-wise error rate (FWER) similarly to CHARM FWER but considering only DMRs containing similar number of probes and selecting for each bootstrapping only those candidate DMRs (cDMRs) that are also significant in a permutation test in order to discard outlier-driven results (see the “Permuted *p* value during bootstrapping” section in the “Methods”; Additional file 3: Methods: Bootstrapping for statistical validation). The original methodology is described in the CHARM package [27]. Briefly, FWER is computed for each DMR and for each statistic; in the case of *avg* it is computed as the proportion of maximum *avg* (across all iterations) that is greater than or equal to the *avg* of the DMR (similarly for *max* and *area.raw*). In both TS1 and TS2 the resampling was conducted 2000 times.

### Permuted *p* value during bootstrapping

We observed that many candidates were selected based on locus-specific outliers, i.e., a unique twin pair with extreme differences in the studied DMR but not showing differences globally and therefore not discarded as a sample. To discard these cDMRs both in the discovery run and for all bootstrapping iterations (to avoid inflation), we computed a permuted *p* value for each DMR. For this we first computed for each sample the average (maximum) methylation over the DMR and then by randomizing the labels within each pair of twins we computed an average-associated permuted *p* value (maximum associated permuted *p* value). We excluded cDMRs in both the discovery run and bootstrapping iterations if the permuted *p* value was >0.1 for both statistics (maximum and average). The number of permutations computed was 32 for TS1 and 128 for TS2; these numbers are limited by the number of samples.

### Functional analyses

Genomic Regions Enrichment of Annotations Tool (GREAT) analysis [29] was done using the web interface provided at Bejerano’s lab (http://bejerano.stanford.edu/great/public/html/). The method defines domains for every gene and then uses this domain to map non-coding *cis*-regulatory regions to genes; each region may be mapped to more than one gene as domains may overlap. The purpose is to perform a functional enrichment at the gene level but overcoming the biases that may be introduced in the mapping of regions to genes; for example, genes in deserts may have larger number of regions associated with them but may not represent regulatory functions. We used GREAT version 2.0.2, with species assembly *hg18* and association rule *Basal + extension: 5,000 bp upstream, 1,000 bp downstream, 1,000,000 bp max extension, curated regulatory domains included*.

### Sorted cell analysis

For cell type-specific methylation profiling, CD4^+^ and CD8^+^ T cells, CD56^+^ NK cells, and neutrophils were isolated from peripheral blood from five healthy male donors (mean age ~38 years) as described in Reinius et al. [30]. CHARM was performed as above.

### Cell proportion estimation

To estimate cell proportion, we adapted the methodology described in [31], originally developed for the Illumina 450 K array. We downloaded software implementing the method from http://people.oregonstate.edu/~housemae/software/. To adapt it to the CHARM array we first identified DMRs for every cell type (e.g., CD4^+^ samples versus rest of samples). We pooled those DMRs (sorted-cell DMRs) and generated a methylation profile for each sorted cell type by averaging the methylation of the probes within the DMRs. We then fit a regression model (“validation model” in the original notation) to select the most informative sorted cell DMRs. Next we solved a quadratic problem (QP) in order to identify the cell proportions within our TS1 and TS2 samples. We added to the QP the condition that the *sum of all the proportions was required to be 1* in order to gain stability in the results. We selected 300 sorted-cell DMRs as informative DMRs.

### Projection analysis

We wanted to check if the identified DMRs in TS1 (considering cell proportion correction) were likely cDMRs in TS2 using TS2 data. To do this we used the genomic intervals (DMRs found in TS1) and computed the permuted *p* value [32] when using data from TS2. To compute the permuted *p* values we used two of the *statistics* used during DMR finding: *max* and *average* (see “Methods”); note that the *area.raw* statistic in this case provides similar results to the *average* statistic. For each statistic and for each TS1 DMR we computed a *score p* value, where *score* can be *max* or *average*. Similarly, we investigated TS2 DMRs in the TS1 data.

### Changes in DMRs versus confounders

Considering the low number of samples, it is not possible to include all covariates directly into the models. Hence, covariates thought to be most relevant were chosen to investigate if the methylation differences identified are associated with any relevant covariate. Four covariates were considered: age, gender, HLA epitope, and smoking. For age and gender, and considering that we are using twin samples, we investigated the association between gender and age, and the “differences in methylation” by linear modeling analysis for each DMR were computed in R. Concerning the HLA epitope and smoking covariates, linear models between methylation profiles and the variables for each DMR were computed. For each linear model the null hypothesis was the slope associated with the covariate being 0.

### Statistical analysis for validation

Significant DMRs were selected to be validated—those associated with genes *PCDHB14* (DMR1), *PCDHB5* (DMR_nc_06), and *EXOSC1* (DMR18). Methylation analysis by bisulfite pyrosequencing was conducted in the CpG sites described in Additional file 2: Table S4. Two types of analyses were performed: technical validation and replication. First a technical validation was done with bisulfite pyrosequencing by analyzing the same individuals profiled in CHARM; the percentage of times methylation differences in twins were in agreement when comparing CHARM and bisulfite pyrosequencing was computed (“Ratio” column in Additional file 2: Table S4). Next a differential methylation analysis was performed by linear modeling using pyrosequencing data (“Technical” column in Additional file 2: Table S4) with and without deconvolution. In all cases the *p* values were not significant, but the directions of the changes (slopes in linear models) in methylation were conserved. For cell-correction analysis we used as covariates the cell proportion estimations computed in CHARM. Finally, using bisulfite pyrosequencing profiling, a new set of individuals were included and the analysis repeated only without deconvolution. Again, no statistically significant associations were found; however, the slope was in the opposite direction to that in the original cohort in Val5 alone.

### Meta-analysis

Meta-analysis on the bisulfite pyro-sequencing data was performed by combining the (unpaired) technical verification and the replication cohort. Additional file 2: Table S4 includes two columns depicting the outcomes of combining the validation and replication samples. We used two different methodologies: (1) *p* value based meta-analysis by the “summation of *p* value” method [33]; and (2), effect size-based meta-analysis considering fixed effects [34] (in all effect size-based analyses residual homogeneity was not rejected, so we used a fixed effect model). Both methodologies provided very similar results, although, as expected, the effect size-based analysis was more powerful.

## Results

### Characteristics of investigated monozygotic twins using CHARM

We analyzed five MZ twin pairs discordant for the presence of ACPA at risk for developing RA (TS1) but without known established RA disease (called “healthy” here) and seven pairs discordant for ACPA-positive RA (TS2) (Table 1). The twins with ACPA-positive RA had varying disease duration with a median of 20 years (range 0–56) and were all treated with disease-modifying anti-rheumatic drugs (DMARDs). Additional detailed information about these samples is presented in Additional file 2: Table S1.

All samples passed the CHARM array quality criteria (see “Methods”). The source of the investigated DNA was whole blood. The analysis of the CHARM arrays was done in two steps. In the first step the analysis was conducted without considering cell proportions. Results from this analysis reflect changes in DNA methylation as a result of phenotype as well as of cell type proportion [30, 31, 35]. This analysis provides a better framework for technical validation of the results because it is not affected by possible errors associated with cell proportion correction methodologies. In the second step, differential methylation analysis was conducted with the computationally predicted information of cell proportion changes (see “Methods”). The first step may be better suited for biomarker discovery, while the second step will provide a basis for hypotheses pertaining to the disease pathology.

### Analyses of cell type-driven differential methylated positions and regions

When considering differential methylation only at the probe level, no single differentially methylated position (DMP) was identified at a FDR of <0.20 when comparing ACPA-positive healthy versus their respective ACPA-negative healthy twin siblings (TS1). Neither did we identify any statistically significant DMP when comparing ACPA-positive RA versus their respective ACPA-negative healthy twin sibling (TS2). These results align with the power analysis done for case-control studies in twins [36]. Volcano plots in Additional file 1: Figure S2a (TS1) and S2b (TS2) show the genome-wide differential methylation at the probe level for the two twin sets. We observed a larger number of hypermethylated CpG sites in the ACPA-positive healthy twin siblings in TS1 (Additional file 1: Figure S2a), while ACPA-positive RA individuals (TS2) show the opposite (Additional file 1: Figure S2b). We did not find statistical evidences of the skewness to be associated with confounders (see the “Quality control for confounders” section in the “Methods”). However, we identified age and gender as relevant covariates to investigate in the candidate DMRs.

To identify DMRs, we defined a high-specificity and low-sensitivity approach, aimed at prioritizing the identification of true positives and minimizing false positives when using the current small number of samples. We estimate the significance of a candidate DMR globally by computing the family-wise error rate (FWER) using an adapted bump-hunter-based algorithm as described by Jaffe et al. [37] (see the “Resampling-based family-wise error rate” section in the “Methods”). Two modifications were made: first, we take into consideration the number of probes of the candidate DMRs (cDMRs) and only include bootstrap-based cDMRs that are significant when computing a permuted *p* value (by randomizing the labels within the twin pairs; see the “Permuted *p* value during bootstrapping” section in the “Methods”) [38]. Our second modification filters out cDMRs that are significant based only on locus-specific outliers, i.e., a unique twin pair with extreme differences in the studied DMR but not showing differences globally and therefore not discarded as a sample.

By employing these strict criteria, one DMR, associated with the protocadherin (PCDH) gene *PCDHB14*, was identified as significant in the TS1 group (Additional file 2: Table S2) after filtering for FWER ≤0.10. The limited number of DMRs is expected in the heterogeneous cell population since all individuals are in fact healthy.

The analysis of TS2, a group where substantial differences in cell type composition is expected, revealed six significant DMRs (Additional file 2: Table S2) after filtering for FWER <0.10. In TS2, another gene in the *PCDH* cluster was found to be differentially methylated (Additional file 1: Figure S3 for *PCDHB5*).

Bisulfite pyrosequencing was used for technical validation of a few selected loci (e.g., *PCDH5* in Additional file 1: Figure S3). None of our technical validations achieved statistical significance, although all changes computed used pyrosequencing data were in the same direction as the CHARM results (we will refer to this as the *change being directionally consistent*; see the “Statistical analysis for validation” section in the “Methods”). For an initial technical validation, we selected cDMRs associated with (FWER <0.20) *COL13A1* (Additional file 1: Figure S4) and *SLITRK2* (Additional file 1: Figure S5) genes because the methylation differences were large enough to be analyzed by pyrosequencing; in both cases the changes were in the same direction as those observed in the CHARM data. Additionally, we performed a technical replication by bisulfite pyrosequencing of DMRs associated with *PCDHB14*, *PCDHB5*, and *EXOSC1* (Additional file 2: Table S4); we selected them based on their biological relevance and in all cases we observed the change is directionally consistent. Finally, we conducted bisulfite pyrosequencing of the same CpGs associated with *PCDHB14*, *PCDHB5*, and *EXOSC1* in independent replication cohorts; in all but one case did we observe changes to be directionally consistent (Additional file 2: Table S4). Finally, we conducted a meta-analysis on the pyrosequencing data by combining the technical validation data and the replication cohort data. For this, we used two different methodologies (see the “Meta-analysis” section in the “Methods”) that returned similar results and between one and three significant CpGs (*p* value <0.05; Additional file 2: Table S4) associated with *PCDHB5* (in TS2) and *PCDHB14* (in TS1).

Several of the TS2 DMRs identified were associated with regions that differentiate the methylation profiles of CD4^+^ T cells and neutrophils (see “Methods” and an example in Additional file 1: Figure S6 for *PCDHB5*). Although important for the disease phenotype, these results point in the direction of substantial methylation changes being due to differences in cell proportion between ACPA-positive RA and healthy individuals, which is consistent with our previous report [2].

### Analysis of phenotype driven differentially methylated regions: comparison with a non-twin cohort

**Fig. 2:**
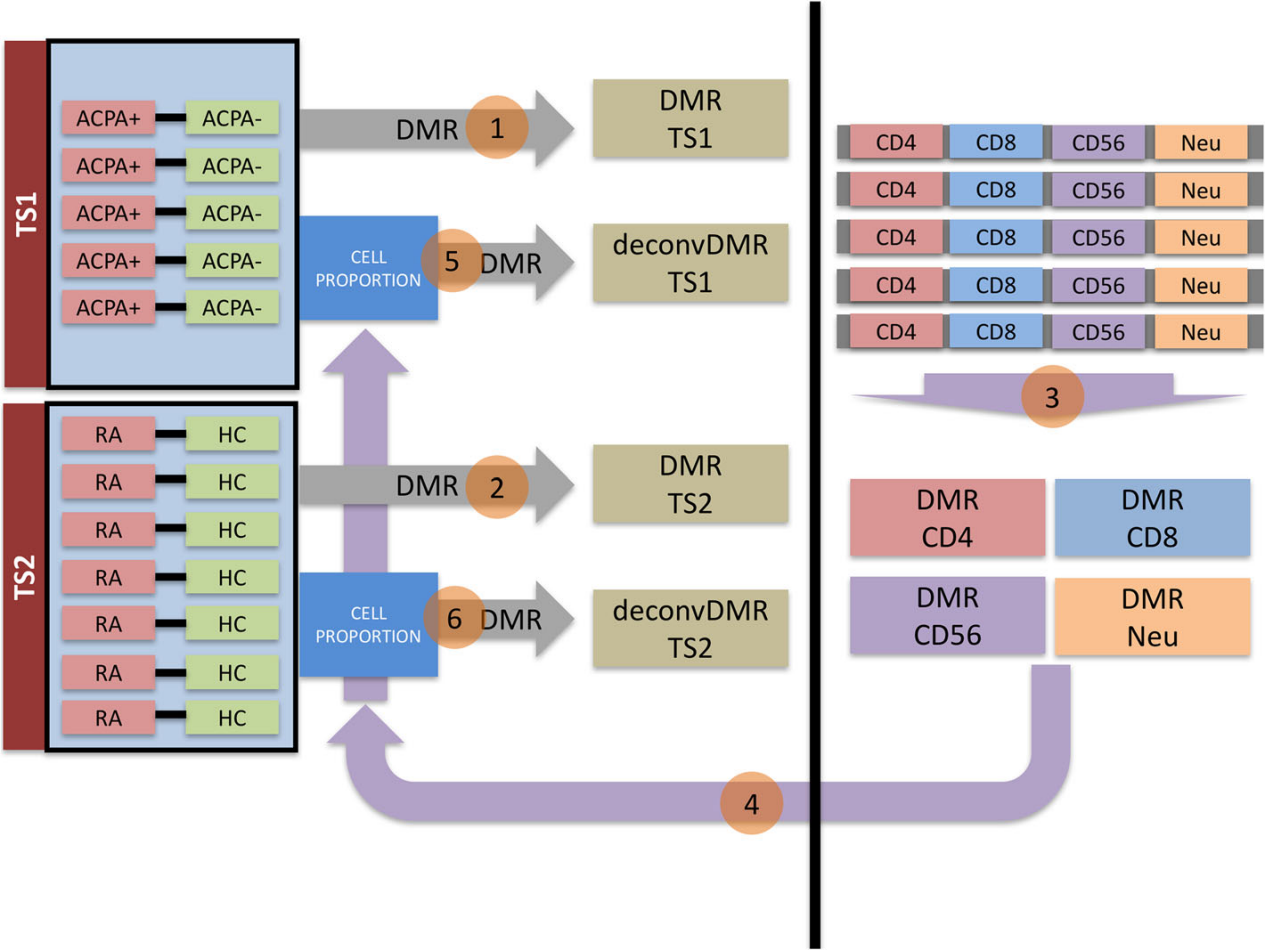
Schematic representation of the analysis. *TS1* and *TS2* denote the tests comparing ACPA-positive healthy twin versus ACPA-negative healthy twin and ACPA-positive RA twin versus ACPA-negative healthy twin, respectively. Steps 1 and 2 denote the first step of the analysis, DMR identification in TS1 and TS2 without cell proportion adjustment. In step 3 we computed DMRs between each pair of cell types (neutrophils, CD4^+^ T cells, CD8^+^ T cells, and CD56^+^ NK cells) and observed that DMRs identified without cell proportion adjustment were associated with cell type, so likely to be associated with changes in cell proportion. For this reason in step 4 we estimated cell proportion in each sample by adapting the method of Houseman et al. [31] (see the “Cell proportion estimation” section in the “Methods”). In step 5 and 6 we used cell proportion estimations as covariates in the identification of DMRs in TS1 and TS2. A DMR is considered statistically significant if it is significant both globally (FWER <0.10) and locally (permuted *p*-value <0.10); details are provided in the “Methods”

In order to identify differentially methylated regions caused by changes in cell type proportion, we repeated the statistical analysis while considering the cell type proportion of each sample (cell type deconvolution/correction). To do this, we used the strategy depicted in Fig. 2. As a first step we analyzed the methylation profile by CHARM in physically sorted CD4^+^ T cells, CD8^+^ T cells, neutrophils, and CD56^+^ NK cells from five healthy individuals. Those profiles allowed us to identify DMRs characteristic of each of these cell types (see the “Sorted cell analysis” in the “Methods”). By combining those DMRs we adapted an existing and validated computational procedure [2, 31] to generate robust estimations of the cell proportions in each sample (see the “Cell proportion estimation” section in the “Methods”). As a second step we applied the same DMR-finder methodology used for the “non cell-corrected analysis” (see the “DMR candidate identification” section in the “Methods”) but this time we included as covariates the estimated cell proportions.

When comparing cell proportions (Table 2) we did not observe statistically significant differences after a *t*-test analysis and after correction for multiple testing, but we did observe larger proportions of neutrophils in most ACPA-positive RA twins (five of seven) compared to their ACPA-negative healthy siblings (TS2), which supports our estimations observed previously [2]. By combining results from [2] and our current data (although not significant) showing the same directionality, we conclude that correction for cell proportion is necessary. We also observed a non-significant decreased population of CD56^+^ NK cells in the ACPA-positive healthy samples in the TS1 group.

**Table 2:**
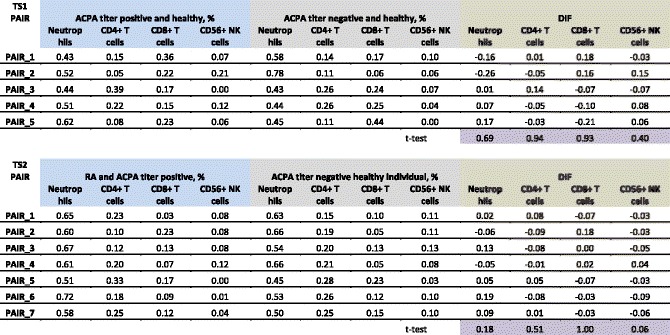
Estimation of cell proportion

For each sample the estimation of neutrophils, CD4^+^ T cells, CD8^+^ T cells, and CD56^+^ NK cells is provided, as described by Houseman et al. [31]. For each twin pair the difference in cell proportion (computed in ratios from 0 to 1, equivalent to percentages) for each cell type was computed in DIF; the purple cells indicate the *p* values from the paired *t*-test comparing differences. The sum of all ratios for a sample may differ from 1 due to rounding in the table; the analysis was performed without rounding

When considering differential methylation only at the probe level after correction for cell proportion, no single DMP was identified at a FDR <0.20 in either TS1 or TS2. Volcano plots in Fig. 1a (TS1) and 1b (TS2) show the genome-wide differential methylation at the individual probe level for the two twin sets. We observed a larger number of hypermethylated CpG sites in both comparisons.

**Fig. 3:**
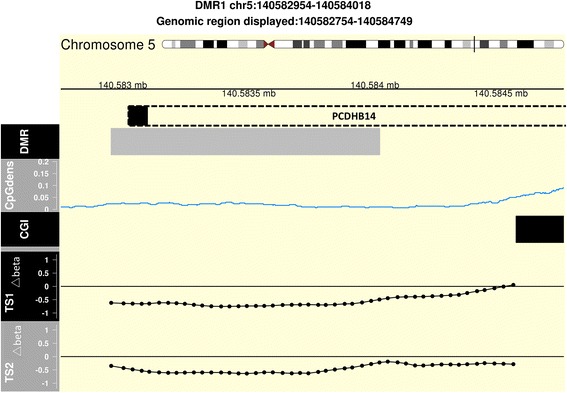
DMR1, from TS1 after cell proportion correction, at the promoter region of *PCDHB14* (chr5 140582954–140584018). *DMR* denotes the DMR location (*grey box*); *CpGdens* denotes CpG density as computed by CHARM [27] (CpG); *CGI* denotes the location of CpG islands (*black box*). *TS1 Δbeta* and *TS2 Δbeta* shows the smoothed linear slope (differences in methylation or *delta*) associated with ACPA-positive healthy and ACPA-positive RA twin, respectively, in the linear model which is used CHARM [27] to identify DMR candidates. Every point denotes a probe location. The location of the gene *PCDHB14* is shown in a *dotted box*; the *black square* on the left denotes the location of the transcription start site

However, DMR analysis in the TS1 comparison after cell type correction returned 17 DMR candidates (FWER ≤0.10; see “Methods”; detailed list in Table 3). Of those, 14 DMRs were found in either CpG shores or CpG islands (Table 4; Fig. 3; Additional file 1: Figure S7–S23).

**Table 3:** DMRs identified after cell type correction

DMR name	Chromosome	Start	End	nprobes	FWER average	FWER maximum	FWER area	Permuted *p* value	Type
DMR1	Chr5	140582954	140584018	31	*<0.01*	*<0.05*	***<0.005***	0.06	TS1
DMR2	Chr11	74630937	74631216	7	*<0.05*	*<0.01*	*<0.1*	0. 03	TS1
DMR3	ChrX	51087402	51089195	32	*<0.1*	*<0.1*	*<0.01*	0. 03	TS1
DMR4	Chr14	54101669	54102157	6	*<0.1*	*<0.1*		0.03	TS1
DMR5	Chr2	85215637	85215811	6	*<0.1*			0.06	TS1
DMR6	Chr14	96038274	96038760	11		*<0.01*		0.06	TS1
DMR7	Chr9	4731364	4731640	6		*<0.05*		0.03	TS1
DMR8	Chr1	198645284	198645642	7		*<0.1*		0.03	TS1
DMR9	Chr1	116184060	116185215	33			*<0.05*	0.06	TS1
DMR10	Chr15	75897924	75898101	6		*<0.05*		0.01	TS1
DMR11	Chr2	171280327	171280711	10	*<0.1*			0.03	TS1
DMR12	Chr16	66835947	66836364	12		*<0.05*		0.09	TS1
DMR13	Chr13	113192663	113193262	8		*<0.1*		0.06	TS1
DMR14	Chr22	19603181	19604444	33			*<0.1*	0.06	TS1
DMR15	Chr17	41620864	41621503	16			*<0.1*	0.06	TS1
DMR16	Chr8	61353541	61354034	15			*<0.1*	0.06	TS1
DMR17	ChrX	113720992	113721797	22			*<0.1*	0.06	TS1
DMR18	Chr10	99200020	99200806	23	*<0.05*	*<0.05*	***<0.005***	<0.01	TS2

DMRs (differentially methylated probes) identified after cell type correction in TS1 and TS2; the double criteria for selection is first a FWER <0.20 in at least one of the three statistics (*italics*) and secondly a permuted *p* value <0.1. “Start” and “End” denote the start and end location of the DMR in the chromosome. “nprobes” denotes the number of probes in the DMR. “FWER average”, “FWER maximum”, and “FWER area” denote the FWER associated with each one of the statistics used (see “Methods”). “Permuted *p* value” denotes the *p* value computed for each DMR locally, comparing each DMR statistic to random permutations of the samples. “Type” denotes if the DMR is associated with TS1 (ACPA/healthy discordant) or TS2 (ACPA-positive RA/healthy discordant). *Bold* highlights DMRs whose FWER was <0.005. Chromosomal locations are based on the hg18 build

**Table 4:** Characterization of DMRs identified after cell type correction

DMR name	Gene (distance to TSS)	Projection	Location: gene	Location: CGI	EIRA
DMR1	PCDHB14 (+224)	**0.02**	Promoter	Shore	No
DMR2	SLCO2B1 (+91266), ARRB1 (+109444)	0.28	Intergenic	CGI/Shore	Yes^a^
DMR3	NUDT10 (−3524)	0.48	Gene body	-	No
DMR4	SAMD4A (−2474)	0.67	Gene body	CGI	Yes +
DMR5	TCF7L1 (+1479), TGOLN2 (+193161)	0.30	Promoter	CGI	Yes ^a^ +
DMR6	PAPOLA (+44)	0.56	Promoter	CGI	Yes ^a^ +
DMR7	AK3 (−275)	0.84	Promoter	CGI	Yes +
DMR8	ZNF281 (+326)	0.89	Promoter	CGI	Yes +
DMR9	NHLH2 (+632)	0.55	Promoter	Shore	Yes
DMR10	LINGO1 (−186249), TBC1D2B (+259036)	0.45	Intergenic	CGI/Shore	No
DMR11	SP5 (+412)	0.83	Promoter	CGI	Yes +
DMR12	PLA2G15 (−592)	0.31	Promoter	CGI/Shore	Yes +
DMR13	TMCO3 (−346)	0.73	Promoter	CGI	Yes +
DMR14	LZTR1 (−62745), CRKL (+2099)	0.13	Extended promoter	Shore	*
DMR15	LRRC37A (−107090), KIAA1267 (−15809)	0.94	Gene body	-	*
DMR16	CA8 (+2720)	0.91	Extended promoter	-	*
DMR17	HTR2C (−3412)	0.20	Gene body	Shore	*
DMR18	EXOSC1 (−4655), ZDHHC16 (+4493)	0.69	Gene body	Shore	No

“Gene” provides information on the closest genes and the distance to the transcription start site (TSS). “Location: gene” and “Location: CGI” denote the locations of the center of the DMR in relation to a gene or a CpG island. “EIRA” is “yes” if the DMR overlaps with a DMP from the EIRA cohort [2] or if it is ^a^located within 1000 bp of one; a plus sign in the EIRA column denotes the same direction of change. “Projection” denotes the permuted *p* value of the DMRs of TS1 and TS2 when using methylation data from TS2 and TS1, respectively (see the “Projection analysis” section in the “Methods”); *p* values in bold are those <0.05. An asterisk denotes that no probes within 500 bp of the DMR are available in the Illumina 450 K array and no comparison is thus possible

From the 17 TS1 DMRs, 13 overlapped with probes present in the Illumina 450 K array; ten of the 13 overlapping DMRs are located within 200 bp of the differentially methylated probes identified after cell correction in our previous study of drug-naïve ACPA-positive RA (the EIRA cohort) using Illumina 450 K analysis [2]. Furthermore, the changes in EIRA were directionally consistent with changes in CHARM analysis at eight of the ten sites. The EIRA cohort is a Swedish population-based case-control study (for more information on this cohort, please visit http://www.eirasweden.se/index1.htm).

One DMR (DMR18; Table 3; Additional file 1: Figure S23) was found when analyzing the TS2 group with cell type proportion correction (FWER ≤0.10). DMR18 spans 23 consecutive probes and is associated with the genes *EXOSC1* and *ZDHHC16* (Tables 3 and 4; Additional file 1: Figure S23). This DMR is hypermethylated in ACPA-positive RA and located in a CpG island shore. *ZDHHC16* is a probable palmitoyltransferase gene and the *EXOSC1* gene codes for a core component of the exosome, highly pertinent for innate immunity. Several proteins of this complex are targets of autoantibodies in patients with autoimmune disease [39]. Interestingly, *EXOSC1* is also a significant DMR in the analysis before adjustment for cell type (Additional file 2: Table S2). The DMR18 was not identified in the EIRA study after cell type adjustment.

Additionally, a cell correction-based technical replication was conducted for the bisulfite pyrosequencing by comparing profiles generated previously for *PCDHB14* (for TS1) and *EXOSC1* (for TS2) with the cell proportion estimated by CHARM (see the “Statistical analysis for validation” in the “Methods”); in all cases the change is directionally consistent but without statistical significance (Additional file 2: Table S4, marked in orange).

### Analysis of TS1 candidate differentially methylated regions in the TS2 group

While cell type correction is necessary for a mechanistic understanding of gene regulation and disease pathology (Additional file 1: Figure S6) [2], the methodology to estimate cell proportions introduces other types of technical variance. Since we have a limited number of paired twins in TS1 and TS2 but larger variation in cell proportion estimates in the disease discordant TS2 group, we hypothesize that cell proportion correction methodology may introduce larger variance in TS2 than in TS1, and therefore we have lower power to identify DMRs (despite higher absolute log-transformed fold change in TS2). Hence, this may be the reason for the limited overlap between TS1 and TS2 after cell type deconvolution. To test this hypothesis and to investigate the possible relationship between the two different phases of disease development, we investigated if TS1 DMRs are significant based on permuted *p* values in the TS2 analysis. A single DMR (DMR1) discovered in the cell type-adjusted healthy ACPA discordant analysis (TS1) is statistically significant in ACPA-positive RA data (TS2) (see the “Projection analysis” section in the “Methods”; indicated in bold in Table 4). DMR1 is located in the promoter of *PCDHB14*, part of the protocadherin beta gene cluster [40]. Additionally and importantly, we observed no TS2 DMR that was significant in the TS1 analysis.

## Discussion

The presence of ACPAs preceding the RA phenotype in conjunction with accessible clinical samples supports the notion of utilizing the pathology of ACPA-positive RA as an autoimmunity disease prototype, facilitating the temporal analysis of the contributions of epigenetic modifications in the context of genes and environment. The current study was therefore designed to elucidate epigenetic factors, albeit not functionally causal, associated with ACPA and the development of ACPA-positive RA that are not directly caused by genetic contributions. The pathogenesis of ACPA-positive RA has strong genetic associations in both the MHC cluster on chromosome 6, where the *HLA* genes of the adaptive immune system reside, as well as non-immune genes. A wealth of information for RA has come from comprehensive genome-wide association studies, and from this it has even been possible to determine the involved individual amino acids in functional domains for antigen presentation [41]. We have previously analyzed how epigenetic factors integrate with genotype by employing DNA methylation profiling in an ACPA-positive RA case-control study [2]. In this way, novel genetic associations with genes were revealed that also associated with specific patterns of DNA methylation. It is also important, however, to separate components of the etiology and pathogenesis of RA from the genetic background. Our results also suggest that the twin approach employed here is useful to neutralize the genetic components. Thus, we did not find any DMRs in the MHC region in the current study, likely due to such neutralization of genetic differences in the discordant MZ twins, supporting the notion that differential methylation in the MHC cluster in RA may actually be completely driven by the genotype. Furthermore, none of the loci in the over 100 previously known genotype-dependent non-*MHC* genes overlaps with our identified DMRs [21]. One of these genes, however, *IRF5*, with known genotype associations with RA, contains a DMR in the non-deconvoluted TS2 group, although no known associated SNPs are in this region. Since this gene has many associated polymorphisms, this may suggest a genetic–epigenetic interaction, which is worth further investigation as discussed below.

Our findings reveal an enrichment towards probe hypomethylation in ACPA-positive RA (*p* value <10e(-9)), in line with Karouzkis et al. [42] and Liu et al. [2]. The CHARM methodology has, however, not previously been used to analyze the RA methylome. Importantly, the CHARM design enables an optimized estimation of DMRs defined by close consecutive probes targeting methylated regions rather than single CpG positions [22]. In addition, DMR methodologies have lower power when applied to the Infinium Illumina 450 K array because the latter design includes many isolated probes yielding DMPs. Furthermore, the CHARM array used here employs a 2.1 million feature array compared to the 480,000 probes (and CpGs) in the Infinium Illumina 450 K array and the recent Infinium Methylation EPIC bead array covering 850,000 probes [43]. A region of differentially methylated CpGs is also a stronger and more robust indicator of altered methylation compared to single CpGs; however, methodologies for DMR power analysis are not yet available while there are methodologies for DMP power estimation [26, 36].

Furthermore, since the cell type distribution in the whole blood cell population was likely to differ between the healthy and affected twin within the pairs, a deconvolution algorithm was applied for CHARM, based on known cell type-specific methylation profiles from four cell types, CD4^+^ and CD8^+^ T lymphocytes, CD56^+^ NK cells, and neutrophils. The deconvolution yields an estimate of the relative distribution of these cell types in order to avoid the discovery of methylation pattern changes driven mostly by changes in differential cell count. It is important to realize that when analyzing non-deconvoluted data, the apparent lack of disease-specific differential methylation in a particular CpG may be due to one disease-affected cell type with hypermethylation in that site and another cell type without this change counteracting the overall methylation alteration. This may create a false negative result. Our results clearly reveal that the analysis of deconvoluted data and unadjusted data answer different questions and show the importance of adjusting for the cell type composition when approaching actual (non-cell type-driven) epigenetic changes within a heterogeneous cell population. However, phenotypic changes of cell type characteristics, regarding methylation, may be partially lost by the deconvolution. In addition, the view of a disease-specific cell population may be valuable for biomarker discovery. In this analysis we cannot interpret whether this differential methylation is due to increases in a certain cell type or a change of cell type distribution in parallel with methylation changes. We did not find significant functional enrichment for the identified genes (see the “Functional analyses” section in the “Methods”).

The current study aimed to reveal novel regions and genes involved in the temporal development of ACPA-positive RA. By including the ACPA-positive discordant healthy twin pair group, we were able to compare two distinct phases in the development of ACPA-positive RA. The twin set discordant for ACPA should be considered as healthy but at increased risk for developing ACPA-positive RA. The exact risk of developing RA is not known and some twins might never develop RA due to additional protective factors or random factors, while others will. Considering the low power observed in TS2, we performed a targeted analysis to investigate if any genomic regions identified in TS1 (TS1 DMRs) were significant in TS2 using TS2 data (see the “Projection analysis” section in the “Methods”). Interestingly, the top DMR (DMR1 in Table 3) discovered in the deconvoluted healthy ACPA-discordant analysis (TS1) was identified as a relevant candidate also in ACPA-positive RA data (TS2) (*p* value = 0.02). This overlapping DMR1 is associated with the *PCDHB14* gene. The overlap could imply the involvement of the associated genes in an ACPA-positive RA disease trajectory. In our analysis, several DMRs associate with *PCDH* genes, both in TS1 and TS2. The relevance of *PCDH* genes in ACPA and RA phenotypes is further emphasized by the significant outcome from the meta analysis of the bisulfite pyrosequencing. The protocadherin family, with over 70 identified genes, are members of the cadherin super family. They are divided into over 50 clustered α-, β-, and γ-*PCDH* genes, all located on chromosome 5, and non-clustered genes scattered in the genome. These transmembrane protein genes are differentially expressed, predominantly in neuronal dendrites, and have been reported to be involved in self/non-self-recognition and self-avoidance [44]. Furthermore, the PCDH18 protein was recently reported as an activation marker of CD8^+^ memory T cells [45]. The *PCDH* gene clusters have a genomic organization similar to B-cell and T-cell receptor gene clusters. This, together with their differential methylation pattern identified in TS1 and TS2, could imply a role in self-recognition and autoimmunity.

The one significant DMR after adjustment for cell type from the ACPA-positive RA discordant twins associates with the *EXOSC1* gene, which codes for a core component of the exosome involved in the processing, controlling, and degrading of RNA and in cytokine regulation and autoimmunity [46]. The exosome has also been shown to be important in the creation of immunoglobulin diversification [47]. Autoantibodies directed towards components of the exosome complex have been identified in sera of patients with idiopathic inflammatory myopathy (IIM), scleroderma, and PM/Scl overlap syndrome [48]. Also, bioinformatic analysis targeting inflammatory bowel disease (IBD), among other diseases, revealed that *EXOSC1* was one of the top upregulated genes associated with the disease [49]. The *EXOSC1* gene deserves further attention and the role of epigenetically regulated gene regions such as enhancers should be investigated. Since the *EXOSC1* DMR was not identified in the healthy ACPA discordant group, it may be specific to fully developed RA rather than a pre-stage. This DMR was not found to replicate the previous non-twin EIRA study (in which the ACPA-positive RA patients were drug-naïve); therefore, we cannot exclude the possibility that it is driven by DMARD therapy (Additional file 2: Table S1). The number of genes which to any extent associated with differential methylation within the pairs in the RA discordant TS2 group are substantially less (and not all overlapping) compared with those found in the EIRA study. Various reasons for this may exist, but at least two main differences stand out regarding experimental design. First and considering the number of samples, our study has lower statistical power than in the EIRA study. Second, the previous study interrogated DMPs using the Illumina 450 K platform and this study employed CHARM to investigate DMRs, and in addition the probes have limited overlap (as shown in our plots of cDMRs). Third, the current study neutralizes any genetic influence on the differential methylation of RA versus non-RA, which the previous study employing a genetically heterogeneous cohort did not do; therefore, we may expect a limited overlap. Finally, the EIRA study comprises treatment-naïve RA patients, again possibly implying an effect of DMARDs in the current study.

Although novel associations of DNA methylation have been implicated in phases of development of ACPA-positive RA, we cannot at this time determine whether this is causally dependent on DNA methylation. To investigate possible confounders for each DMR, we looked into potential associations between changes in DNA methylation with the following covariates: gender, age, smoking, and HLA epitope information (Additional file 2: Table S3). No significant associations were found (*p* value <0.01), although the results showed that age (for TS1) and gender (for TS2) are covariates to be investigated further in larger cohorts. Importantly, our main candidate, *PCDHB14*, is not significantly associated with any covariate.

Our findings do support the notion that DMR1 from the ACPA-positive healthy individuals analysis (and associated with *PCDHB14*) may be associated with onset of ACPA-positive RA, since this DMR could also be found when analyzed in the context of twin pairs discordant for ACPA-positive RA. The discovered genes associated with DMRs found here can be further used for hypothesis generation.

## Conclusions

Here we used a general statistical framework, adapted to empower a low-sample twin design. This new robust framework was applied to the DNA methylome from two small sets of MZ twins discordant for ACPAs but healthy and ACPA-positive RA, respectively. The unique material for the data represents different phases during the progression of RA, thus enabling us for the first time to interrogate the temporal contribution of epigenetic factors dissociated from genetics to the evolution of the disease. This design made it possible to delineate candidate genes of relevance for development of ACPA-positive RA. The DMR associated with a *PCDH* gene suggests a temporal epigenetic connection between ACPA-positivity and clinical RA. Our results should be of interest for further research in the clinical autoimmune field for hypothesis generation, as well as for the wider research community employing the proposed statistical approach.

## Additional files

Additional file 1: Supplementary **Figures S1** to **S26**. (ZIP 7 mb)
Additional file 2: Supplementary **Tables S1** to **S4**. (ZIP 31 kb)
Additional file 3: Supplementary methods. The file includes extended description of aspects of the bioinformatic analysis. (DOCX 25 kb)
