# Supplementary material for: High-specificity bioinformatics framework for epigenomic profiling of discordant twins reveals specific and shared markers for ACPA and ACPA-positive rheumatoid arthritis
Source: Genome Med. 2016 Nov 22;8:124. doi: 10.1186/s13073-016-0374-0 (PMC5120506; doi:10.1186/s13073-016-0374-0)
Supplement: Additional file 1: — Supplementary Figures S1 to S26. (ZIP 7 mb) [file 13073_2016_374_MOESM1_ESM.zip › 13073_2016_374_MOESM1_ESM.docx]

**Supplemental Figure Legends.**

**Genome-wide epigenetic profiling of monozygotic twins discordant for ACPA or ACPA-positive rheumatoid arthritis reveals novel associated genes.**

**Supplemental Figure Legends**

**Supp. Figure 1. CHARM: distribution of probes.** The plot was generated with ChIPseeker Bioconductor package [^1^](#_ENREF_1). The probes are characterized based on their overlapping with gene locations. Promoters are considered as the genomic region covering 1300 upstream and downstream from the Transcription Starting Site.

**Supp. Figure 2. Volcano plot.** **Differential Methylation summary without considering cell proportion correction**. Log-transformed p-value (y-axis) versus log-transformed Fold Change from the associated linear model. A negative (positive) logFC denotes hypomethylation (hypermethylation) in TS1 and TS2 contrasts; the logFC was computed by using M values (see MM) in a linear model. The horizontal and vertical lines are arbitrary thresholds selected to highlight possible tendencies. (a) Provides the results from ACPA positive healthy vs ACPA negative healthy twin siblings (TS1). (b) Provides the results from ACPA positive RA vs ACPA negative healthy twin siblings (TS2).

**Supp. Figure 3. PCDHB5 associated DMR from TS2 analysis without cell proportion correction (chr5:140494633-140495503).** Upper panel describes the DMR methylation profile as obtained from the CHARM experiment; the top panel shows the levels of methylation for individual probes for ACPA positive RA twin (red) and ACPA negative healthy twin (blue); lines denote the average methylation profile within each group. Middle panel shows the individual (points) and average (line) differences in methylations between each twin pair. The bottom panel presents the DMR location. Lower panel CHARM analysis and pyrosequencing data for validation are compared. The two top panels are zooming in over what is shown in top two upper panels; the two bottom panels provide similar information but for pyro-sequencing validation experiments.

**Supp. Figure 4. COL13A1 associated TS2 without cell proportion correction DMR (chr10:71229788-71230625).** The upper plot describes the DMR methylation profile as obtained from CHARM experiment. In the upper plot the top panel shows the levels of methylation for individual probes for ACPA positive RA twin (red) and ACPA negative healthy twin (blue); lines denote the average methylation profile within each group. Middle panel shows the individual (points) and average (line) differences in methylations between each twin pair. The bottom panel presents the DMR location. In the bottom plot the CHARM analysis and pyrosequencing data for validation are compared. The two top panels are zooming in over what is shown in top two upper panels; the two bottom panels provide similar information but for pyrosequencing validation experiments.

**Supp. Figure 5. SLITRK2 associated TS2 without cell proportion correction DMR (chrX:144705098-144705656)**. The upper plot describes the DMR methylation profile as obtained from CHARM experiment. In the upper plot the top panel shows the levels of methylation for individual probes for ACPA positive RA twin (red) and ACPA negative healthy twin (blue); lines denote the average methylation profile within each group. Middle panel shows the individual (points) and average (line) differences in methylations between each twin pair. The bottom panel presents the DMR location. In the bottom plot the CHARM analysis and pyrosequencing data for validation are compared. The two top panels are zooming in over what is shown in top two upper panels; the two bottom panels provide similar information but for pyrosequencing validation experiments.

**Supp. Figure 6. Association of PCDHB5 DMRs (Supp. Fig. 3) in TS2 (without cell-proportion correction) to cell-specific methylation markers.** (1) ESTIMATED B VALUE provides the percentage of methylation for every individual (dots) and the average for every clinical group (line). Every vertical group of points denote the position of a probe. (2) DMR: Location of *TS2* DMR obtained without cell proportion correction. (3) CGI: CpG island location (black box).

**Supp. Figure 7-23. DMR depicted from DMR2 to DMR18 (See Table 3 and 4).** Description is similar to that in Figure 3. DMR: denotes DMR location (grey box); CpGdens denotes CpG density as computed by CHARM [^2^](#_ENREF_2) (CpG); CGI: denotes the location of CpG islands (black box); EIRA: location of significantly differentially methylated probes after cell-proportion correction in [^3^](#_ENREF_3) (blue box with red borders). TS1 △beta (TS2 △beta) shows the smoothed linear slope (differences in methylation or *delta*) associated to ACPA positive healthy (ACPA positive RA twin) in the linear model which is used charm [^2^](#_ENREF_2) to identify DMR candidates. Every point denotes a probe location.

**Supp. Figure 24. Analysis of the statistics used in the dmrFind: area.raw.** The figure shows that the area.raw statistic is dependent of the number of probes in the DMR candidate regions, therefore selecting those with larger number of probes. In each plot the grey area denotes the density function of the boot-strapping-generated DMR-area.raw values; red points denote area.raw values of the candidate DMR regions found in the data.

**Supp. Figure 25. Analysis of the statistics used in the dmrFind: max and avg.** The figure shows the max (left plots) and avg (right plots) statistics for randomly generated DMRs (in grey) and for candidate DMR regions (red) for different selection of DMRs based on their number of probes.

**Supp. Figure 26. Quality Control: Multi-Dimensional Scaling (MDS).** The figure depicts the MDS plots for TS1 (panel (a)) and TS2 (panel (b)) samples. In each case the same MDS plot is depicted with labels associated to Age (top-left), Gender (top-right) and Twin Pair (bottom-left). None of those variables are identified as significant confounder.

**References**

1. Yu, G., Wang, L.G., and He, Q.Y. (2015). ChIPseeker: an R/Bioconductor package for ChIP peak annotation, comparison and visualization. Bioinformatics 31, 2382-2383.

2. Aryee, M.J., Wu, Z., Ladd-Acosta, C., Herb, B., Feinberg, A.P., Yegnasubramanian, S., and Irizarry, R.A. (2011). Accurate genome-scale percentage DNA methylation estimates from microarray data. Biostatistics 12, 197-210.

3. Liu, Y., Aryee, M.J., Padyukov, L., Fallin, M.D., Hesselberg, E., Runarsson, A., Reinius, L., Acevedo, N., Taub, M., Ronninger, M., et al. (2013). Epigenome-wide association data implicate DNA methylation as an intermediary of genetic risk in rheumatoid arthritis. Nat Biotechnol 31, 142-147.
