# Supplementary material for: High-specificity bioinformatics framework for epigenomic profiling of discordant twins reveals specific and shared markers for ACPA and ACPA-positive rheumatoid arthritis
Source: Genome Med. 2016 Nov 22;8:124. doi: 10.1186/s13073-016-0374-0 (PMC5120506; doi:10.1186/s13073-016-0374-0)
Supplement: Additional file 1: — Supplementary Figures S1 to S26. (ZIP 7 mb) [file 13073_2016_374_MOESM1_ESM.zip › 13073_2016_374_MOESM1_ESM.pdf]

# Supp. Figures

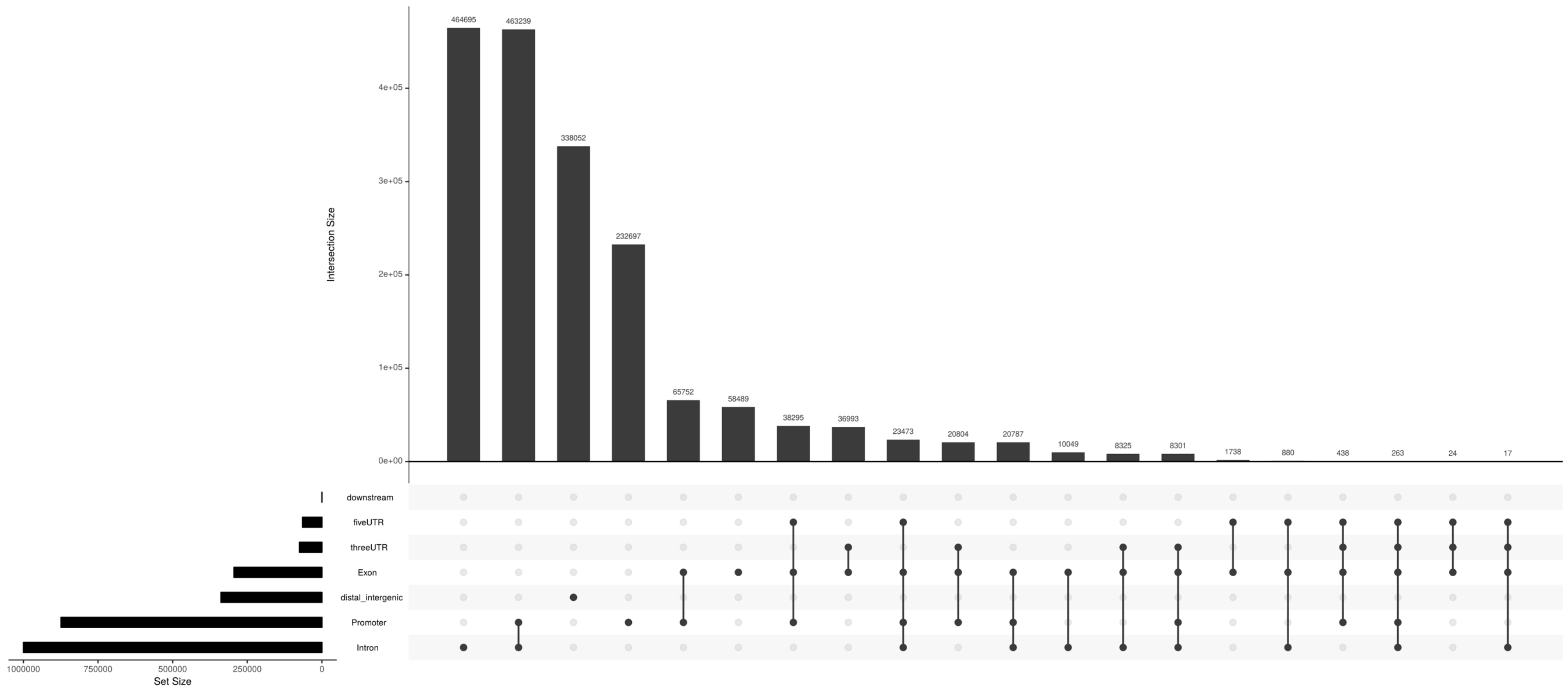

464695
463239
338052
232697
65752
58489
38295
36993
23473
20804
20787
10049
8325
8301
1738
880
438
263
24
17
4e+05
3e+05
2e+05
1e+05
0e+00
Intersection Size
downstream
fiveUTR
threeUTR
Exon
distal_intergenic
Promoter
Intron
1000000
750000
500000
250000
0
Set Size


Supp. Fig. 1

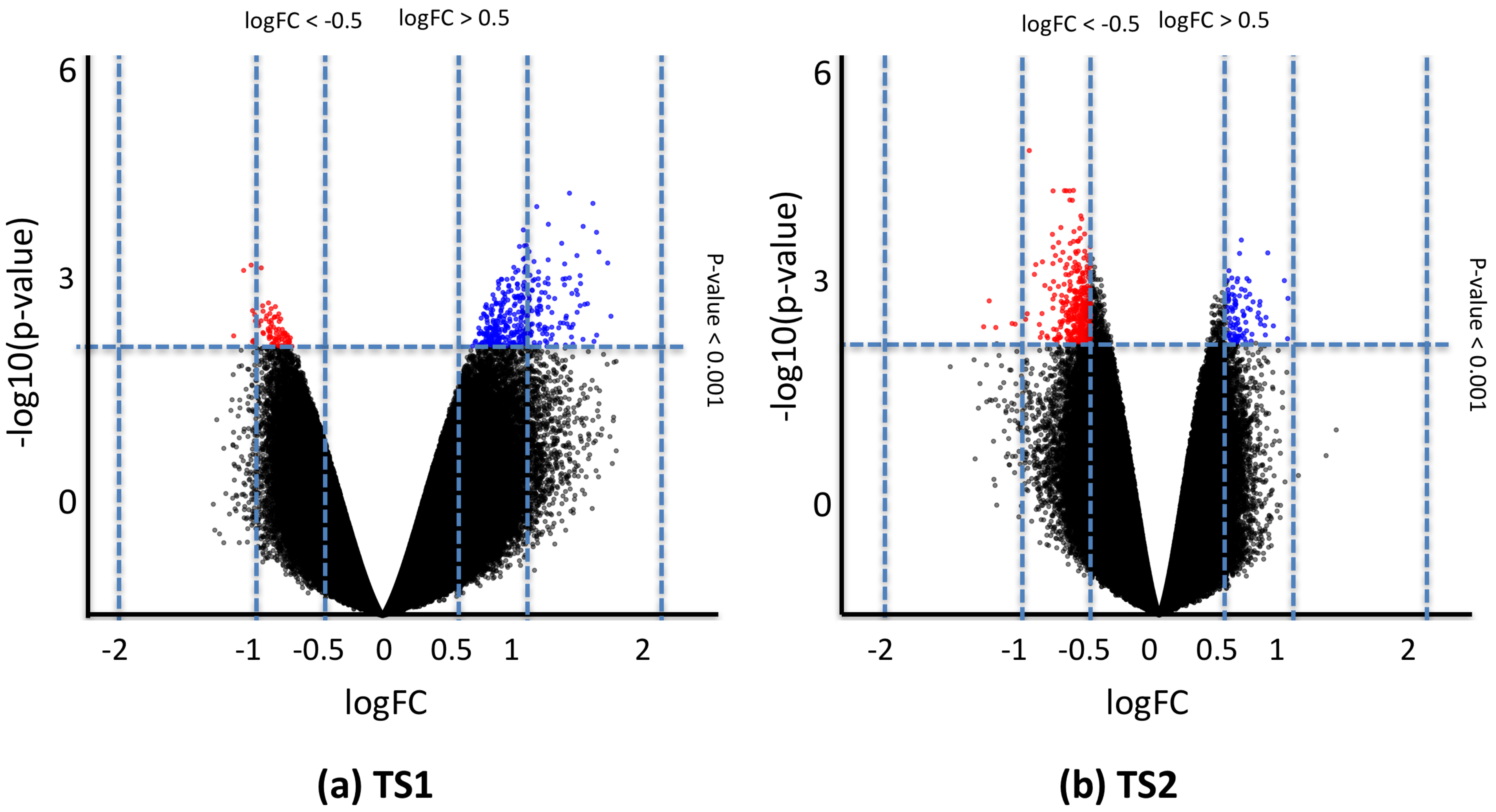
logFC < -0.5
logFC > 0.5
6
3
0
-log10(p-value)
P-value < 0.001
-2
-1
-0.5
0
0.5
1
2
logFC
(a) TS1
logFC < -0.5
logFC > 0.5
6
3
0
-log10(p-value)
P-value < 0.001
-2
-1
-0.5
0
0.5
1
2
logFC
(b) TS2

Supp. Fig. 2

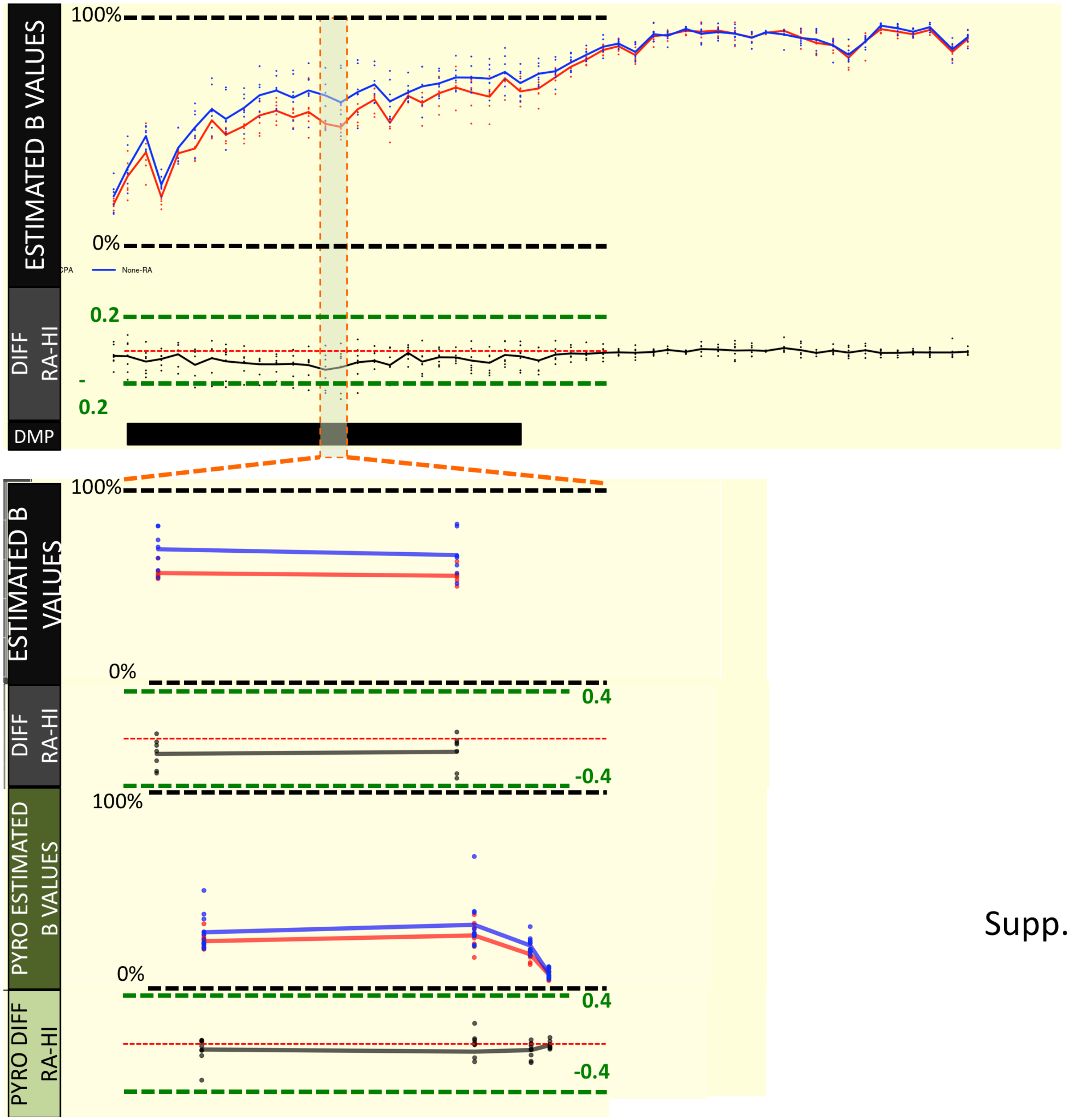
ESTIMATED B VALUES
100%
0%
CPA
None-RA
DIFF RA-HI
0.2
-0.2
DMP
ESTIMATED B VALUES
100%
0%
0.4
DIFF RA-HI
-0.4
PYRO ESTIMATED B VALUES
100%
0%
0.4
PYRO DIFF RA-HI
-0.4

Supp. Fig. 3

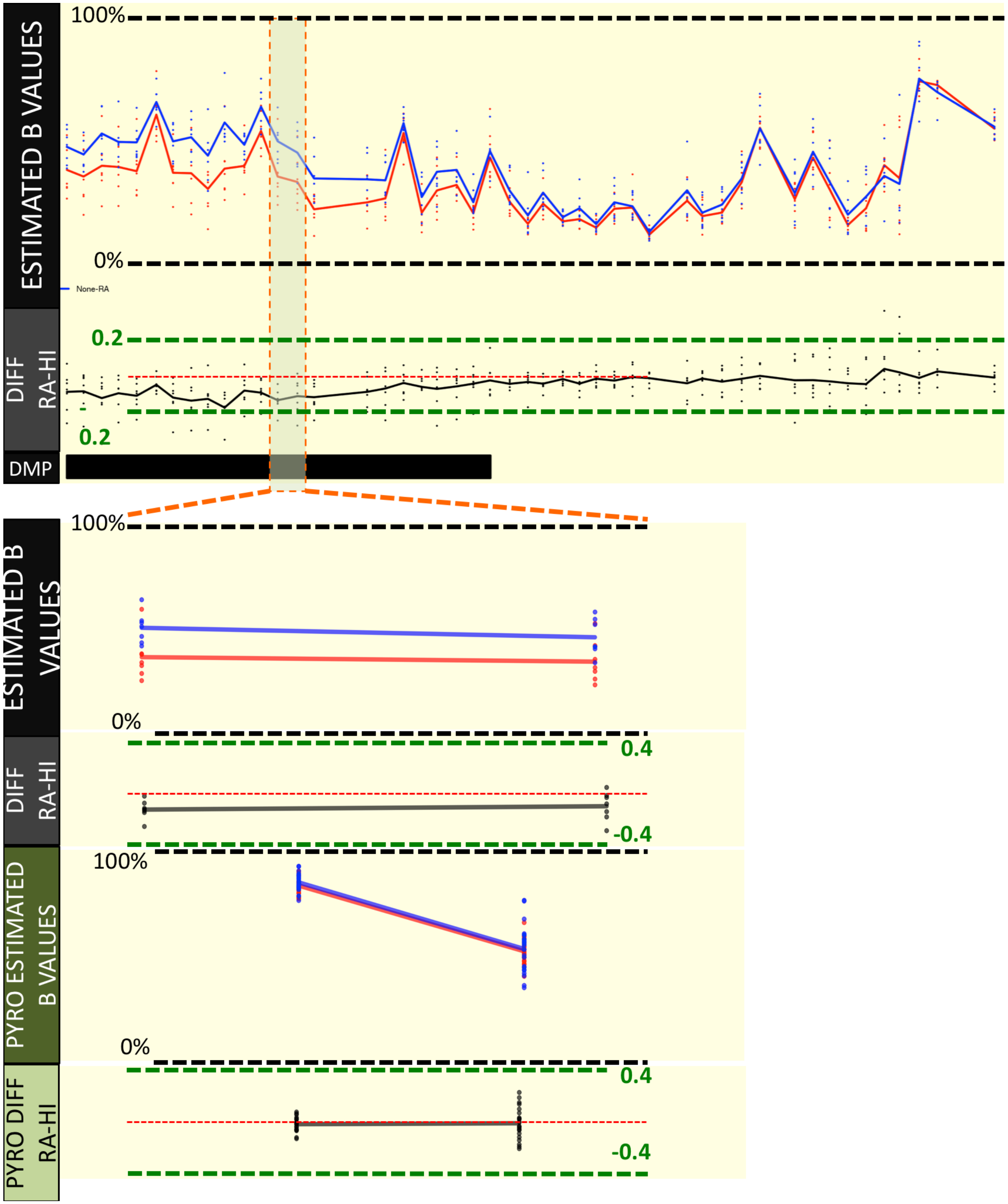
ESTIMATED B VALUES
100%
0%
None-RA
DIFF RA-HI
0.2
0.2
DMP
100%
ESTIMATED B VALUES
0%
0.4
DIFF RA-HI
-0.4
100%
PYRO ESTIMATED B VALUES
0%
0.4
PYRO DIFF RA-HI
-0.4

Supp. Fig. 4

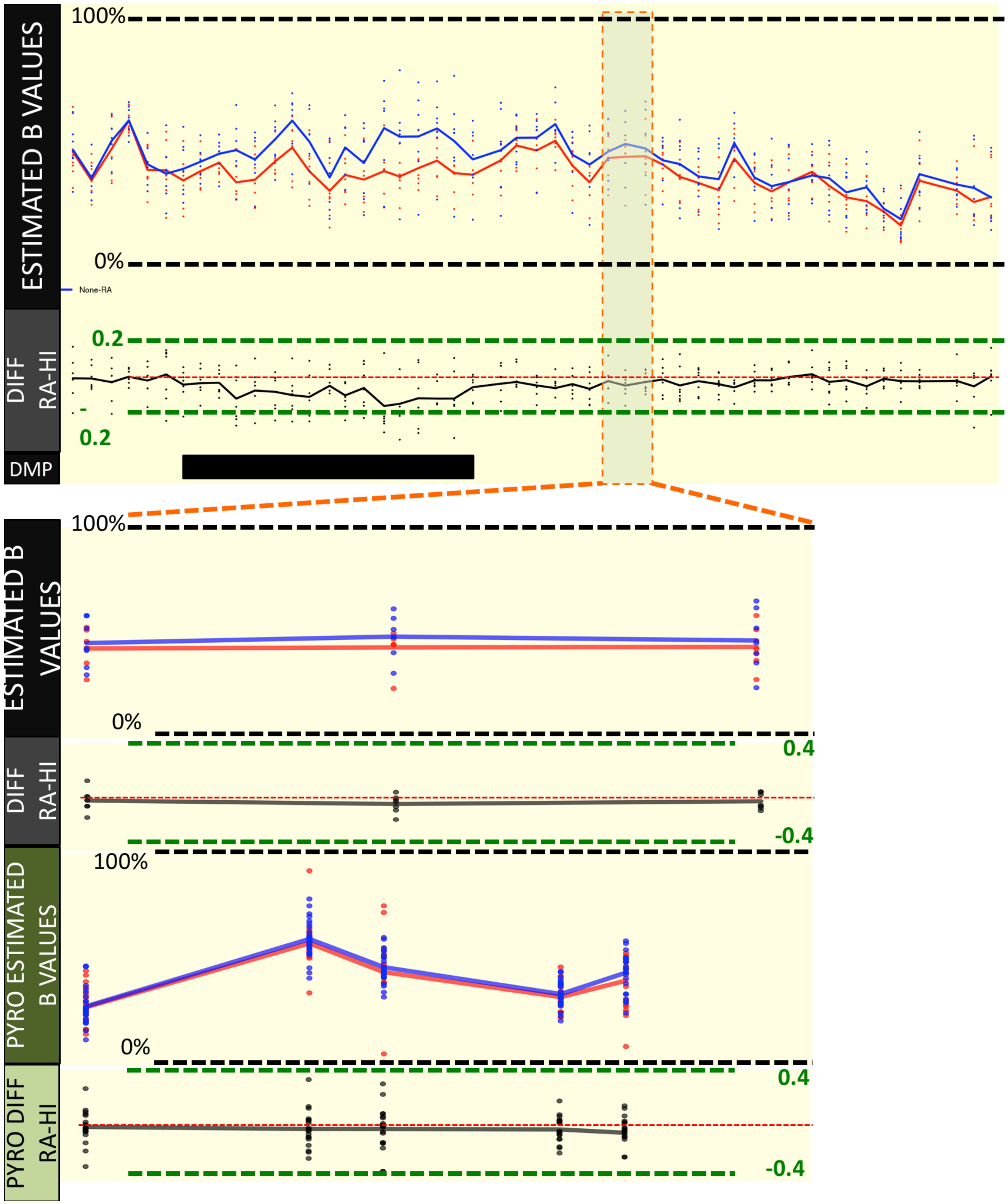

ESTIMATED B VALUES
100%
0%
None-RA
DIFF RA-HI
0.2
0.2
DMP
ESTIMATED B VALUES
100%
0%
DIFF RA-HI
0.4
-0.4
PYRO ESTIMATED B VALUES
100%
0%
PYRO DIFF RA-HI
0.4
-0.4


Supp. Fig. 5

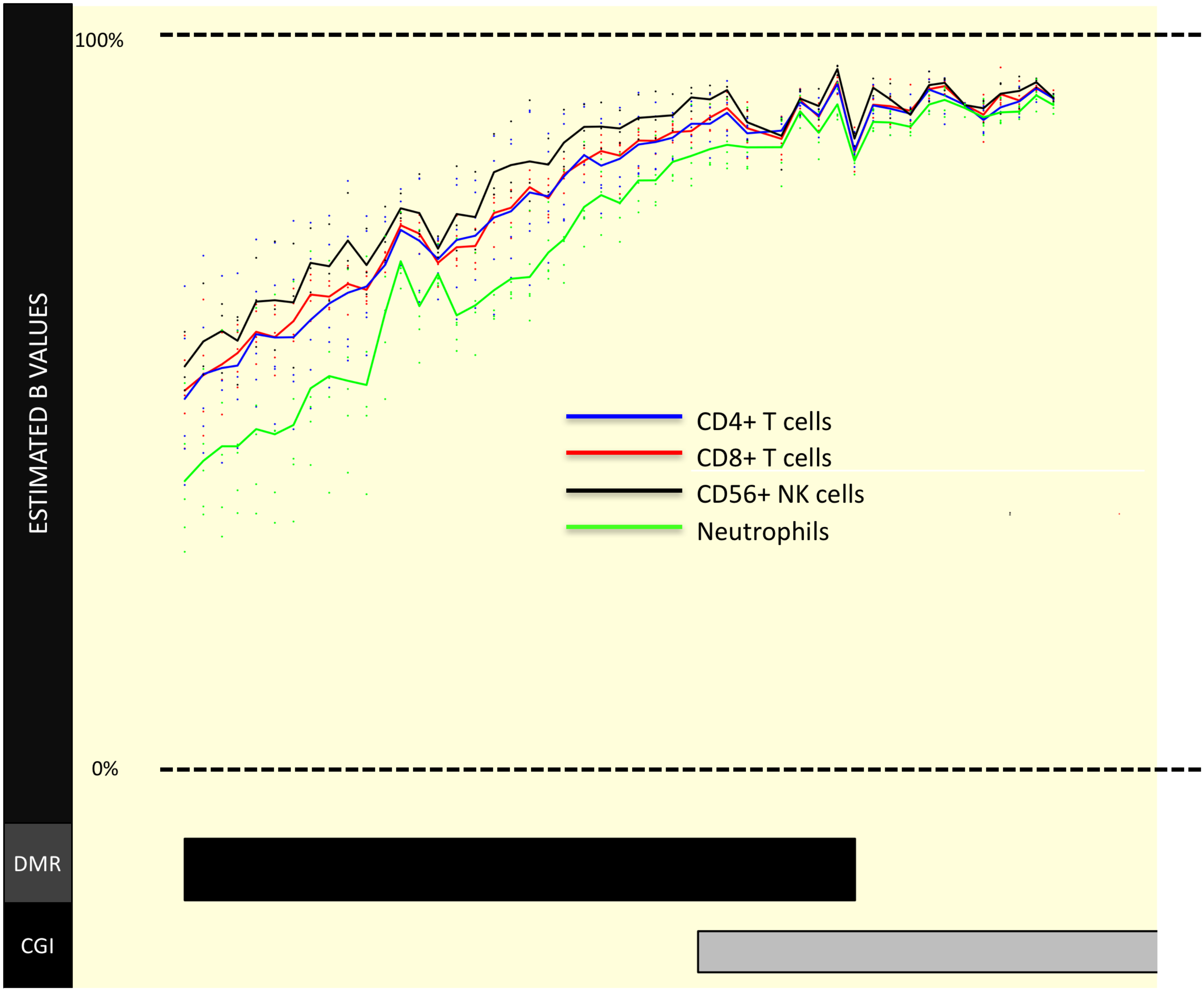
ESTIMATED B VALUES
100%
0%
CD4+ T cells
CD8+ T cells
CD56+ NK cells
Neutrophils
DMR
CGI

Supp. Fig. 6

Supp. Fig. 7

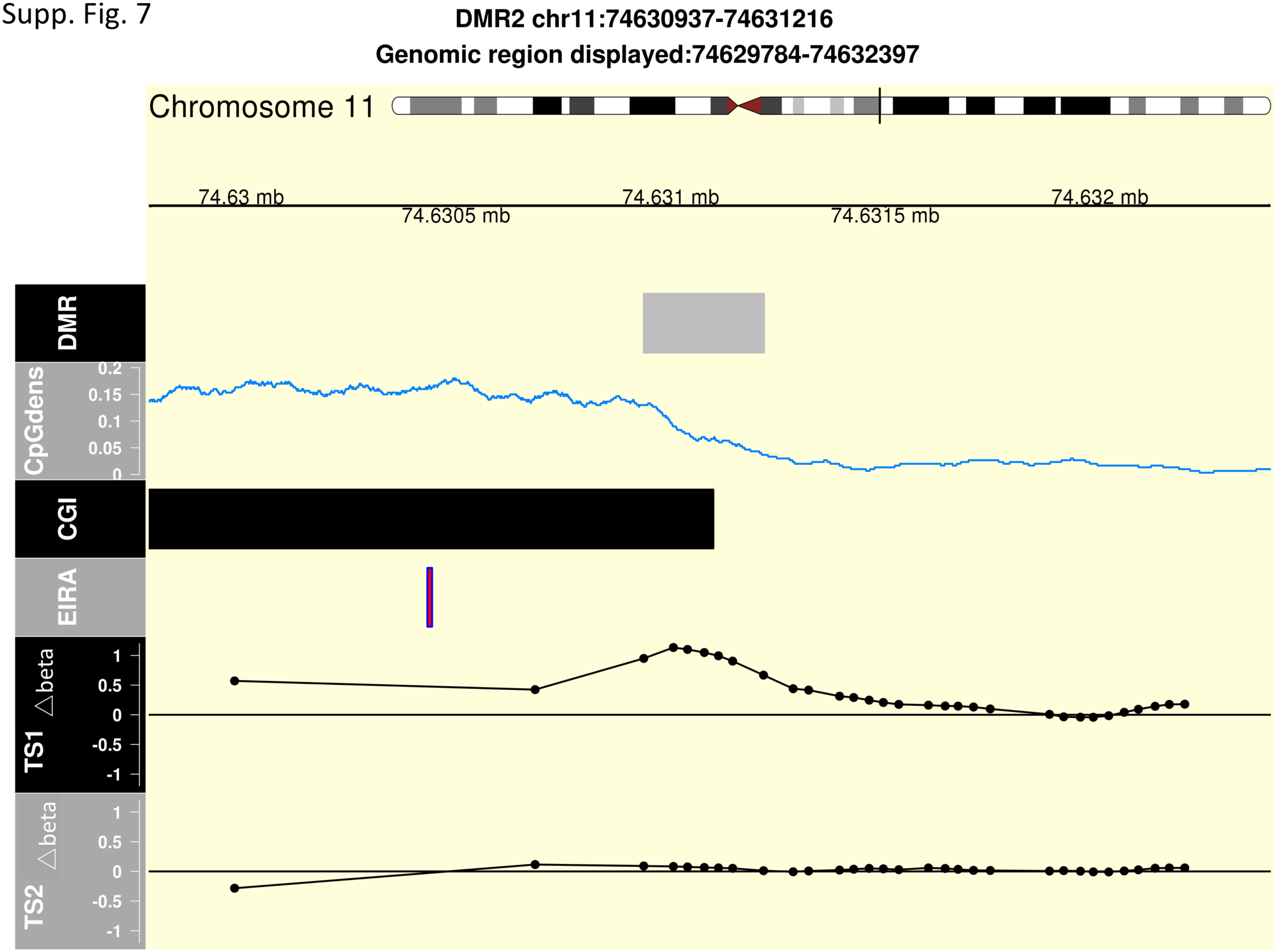

DMR2 chr11:74630937-74631216
Genomic region displayed:74629784-74632397
Chromosome 11
74.63 mb
74.6305 mb
74.631 mb
74.6315 mb
74.632 mb
DMR
CpGdens
0.2
0.15
0.1
0.05
0
CGI
EIRA
TS1
△beta
1
0.5
0
-0.5
-1
TS2
△beta
1
0.5
0
-0.5
-1

Supp. Fig. 8

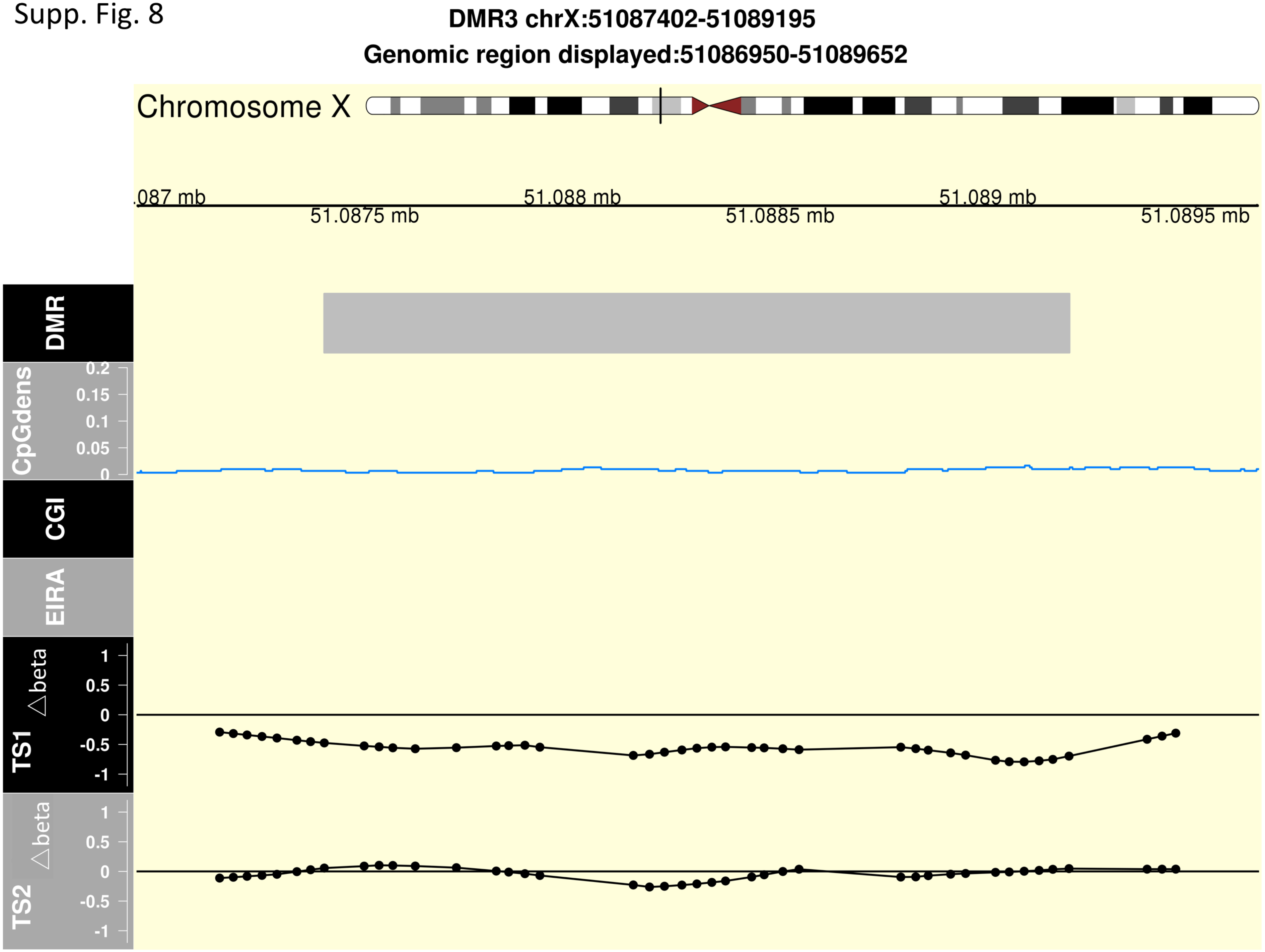

DMR3 chrX:51087402-51089195
Genomic region displayed:51086950-51089652
Chromosome X
.087 mb
51.0875 mb
51.088 mb
51.0885 mb
51.089 mb
51.0895 mb
DMR
CpGdens
0.2
0.15
0.1
0.05
0
CGI
EIRA
TS1
△beta
1
0.5
0
-0.5
-1
TS2
△beta
1
0.5
0
-0.5
-1

Supp. Fig. 9

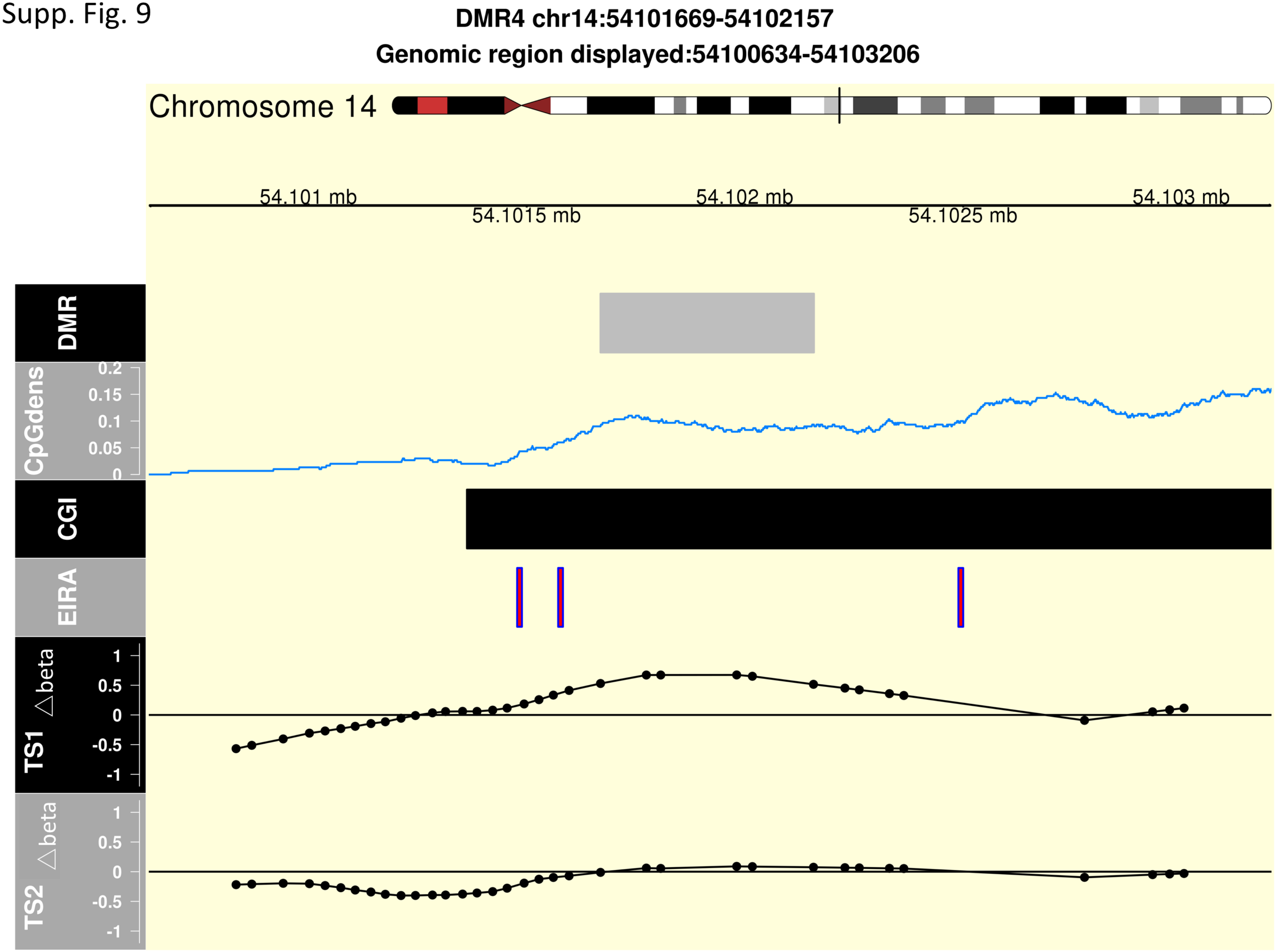

DMR4 chr14:54101669-54102157
Genomic region displayed:54100634-54103206
Chromosome 14
54.101 mb
54.1015 mb
54.102 mb
54.1025 mb
54.103 mb
DMR
CpGdens
0.2
0.15
0.1
0.05
0
CGI
EIRA
TS1
△beta
1
0.5
0
-0.5
-1
TS2
△beta
1
0.5
0
-0.5
-1

Supp. Fig. 10

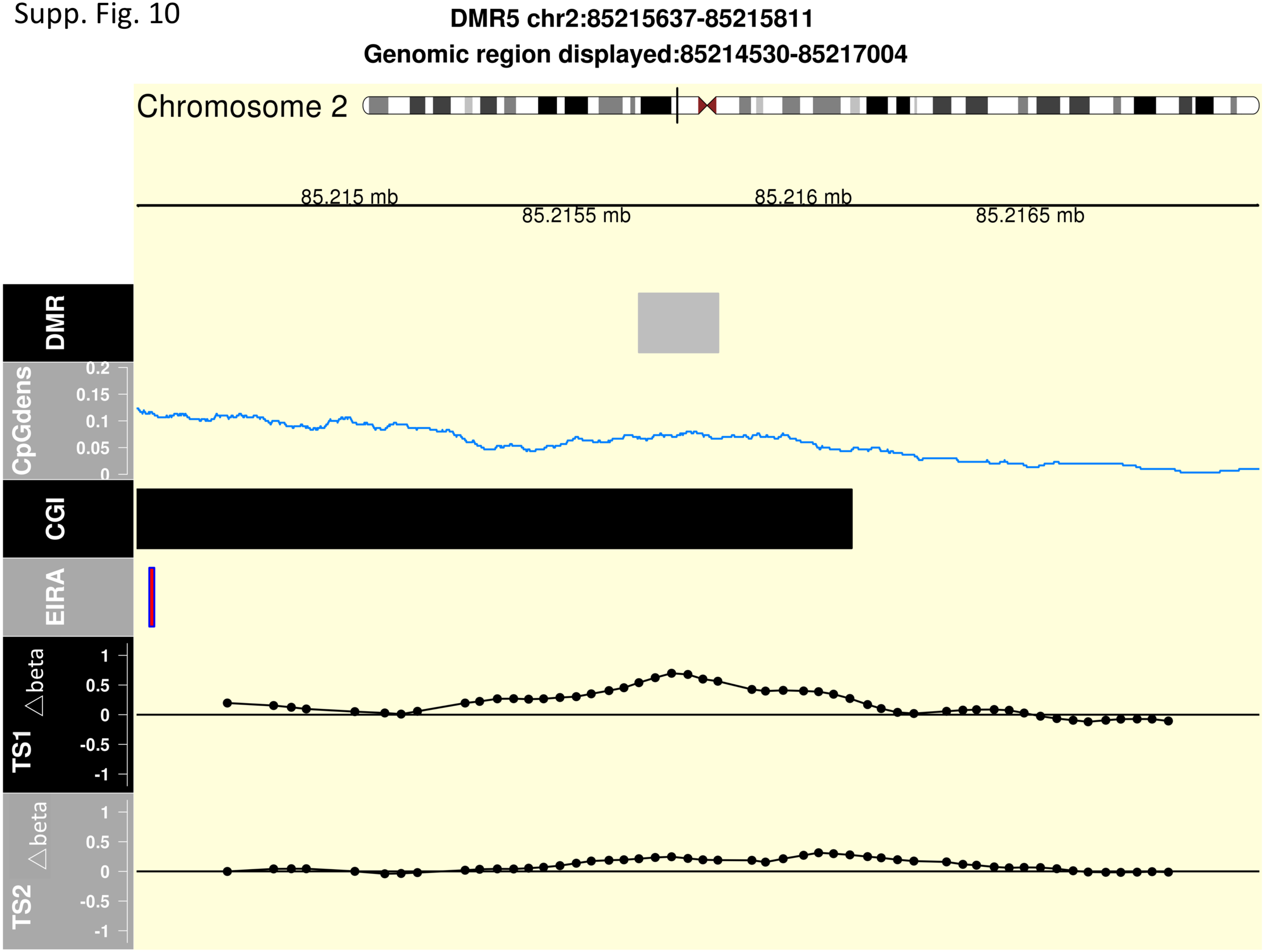

DMR5 chr2:85215637-85215811
Genomic region displayed:85214530-85217004
Chromosome 2
85.215 mb
85.2155 mb
85.216 mb
85.2165 mb
DMR
CpGdens
0.2
0.15
0.1
0.05
0
CGI
EIRA
TS1
△beta
1
0.5
0
-0.5
-1
TS2
△beta
1
0.5
0
-0.5
-1

Supp. Fig. 11

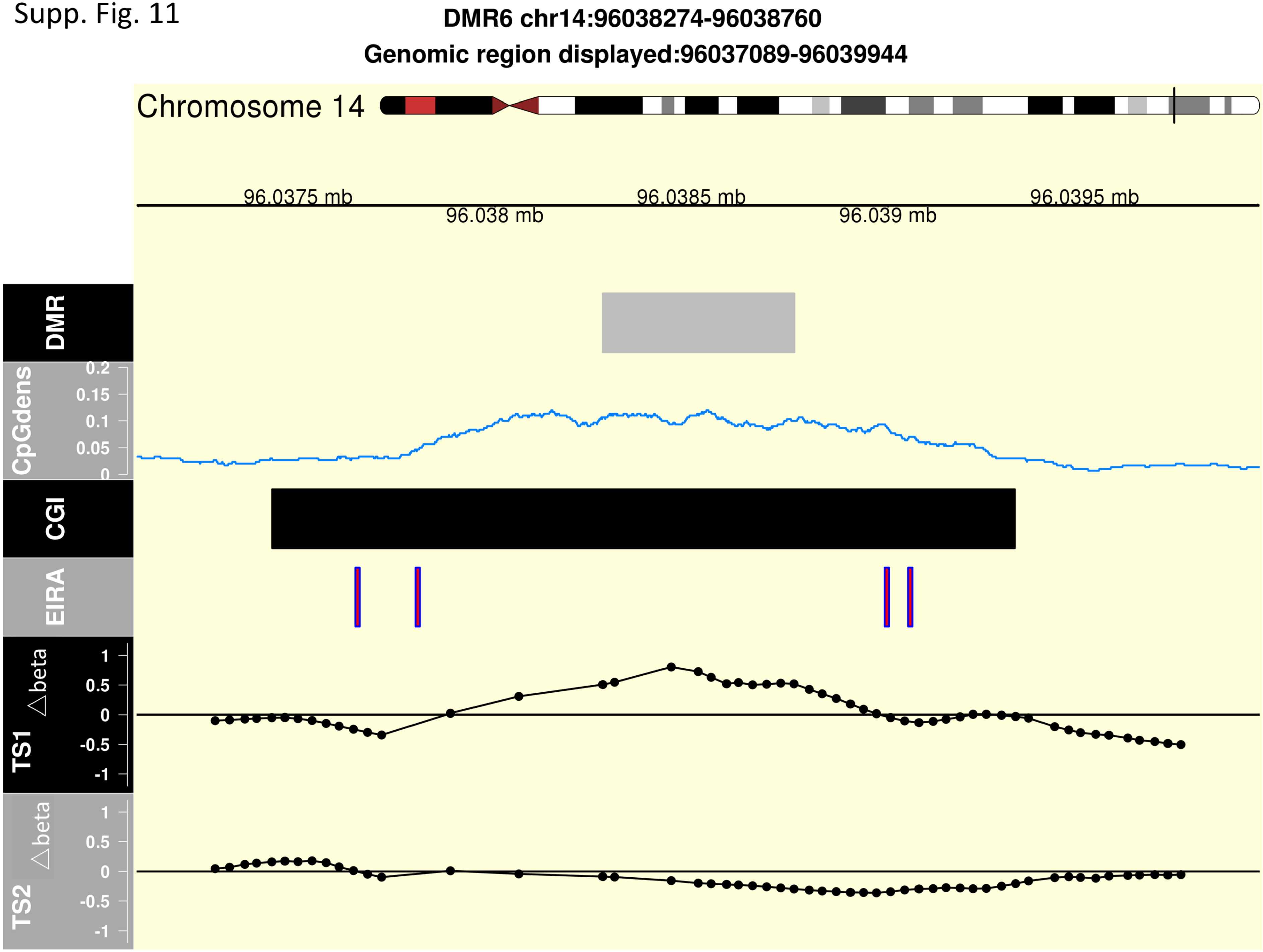

DMR6 chr14:96038274-96038760
Genomic region displayed:96037089-96039944
Chromosome 14
96.0375 mb
96.038 mb
96.0385 mb
96.039 mb
96.0395 mb
DMR
CpGdens
0.2
0.15
0.1
0.05
0
CGI
EIRA
TS1
△beta
1
0.5
0
-0.5
-1
TS2
△beta
1
0.5
0
-0.5
-1

Supp. Fig. 12

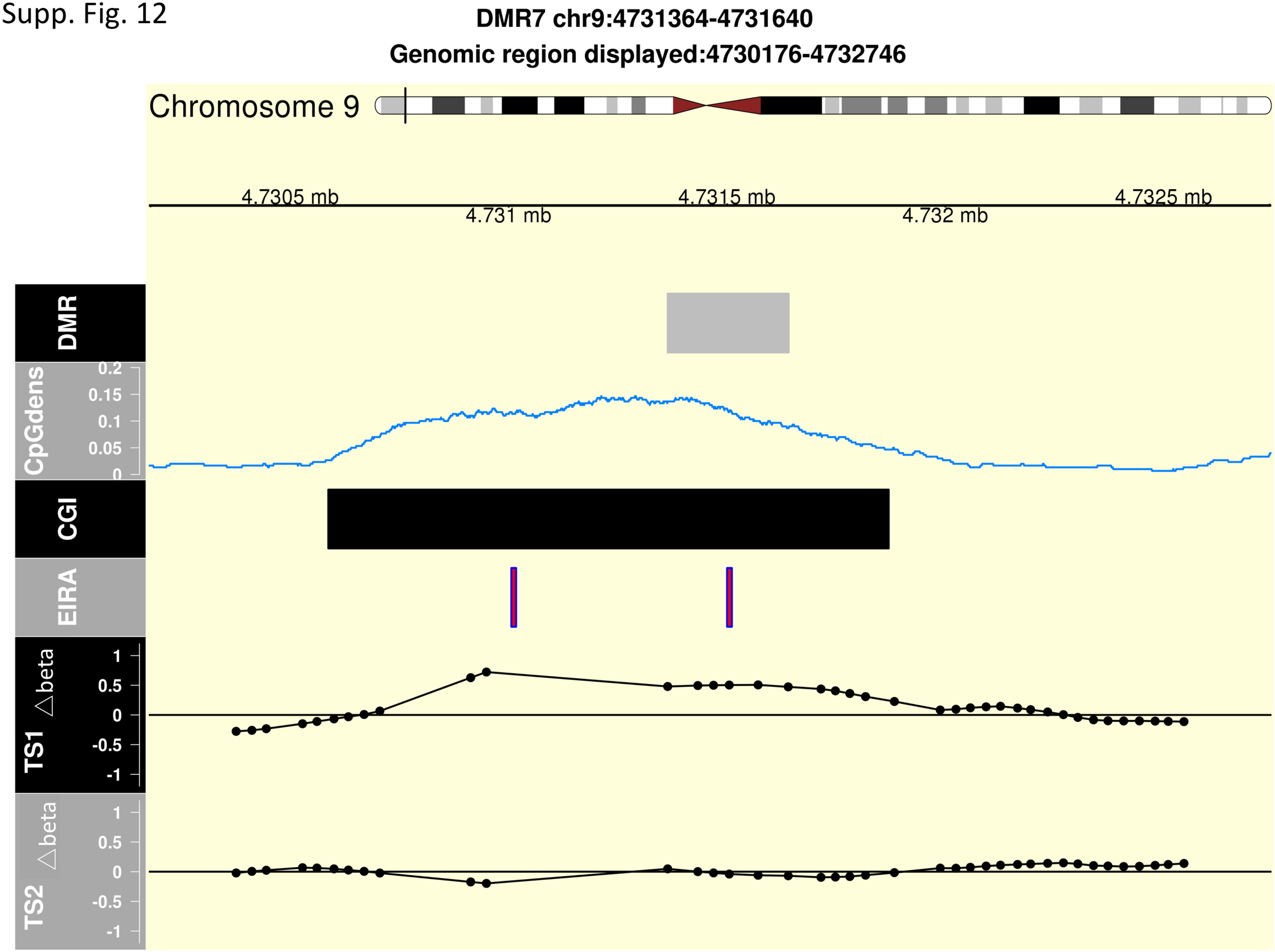
DMR7 chr9:4731364-4731640
Genomic region displayed:4730176-4732746
Chromosome 9
4.7305 mb
4.7315 mb
4.7325 mb
4.731 mb
4.732 mb
DMR
CpGdens
0.2
0.15
0.1
0.05
0
CGI
EIRA
TS1 △beta
1
0.5
0
-0.5
-1
TS2 △beta
1
0.5
0
-0.5
-1

Supp. Fig. 13

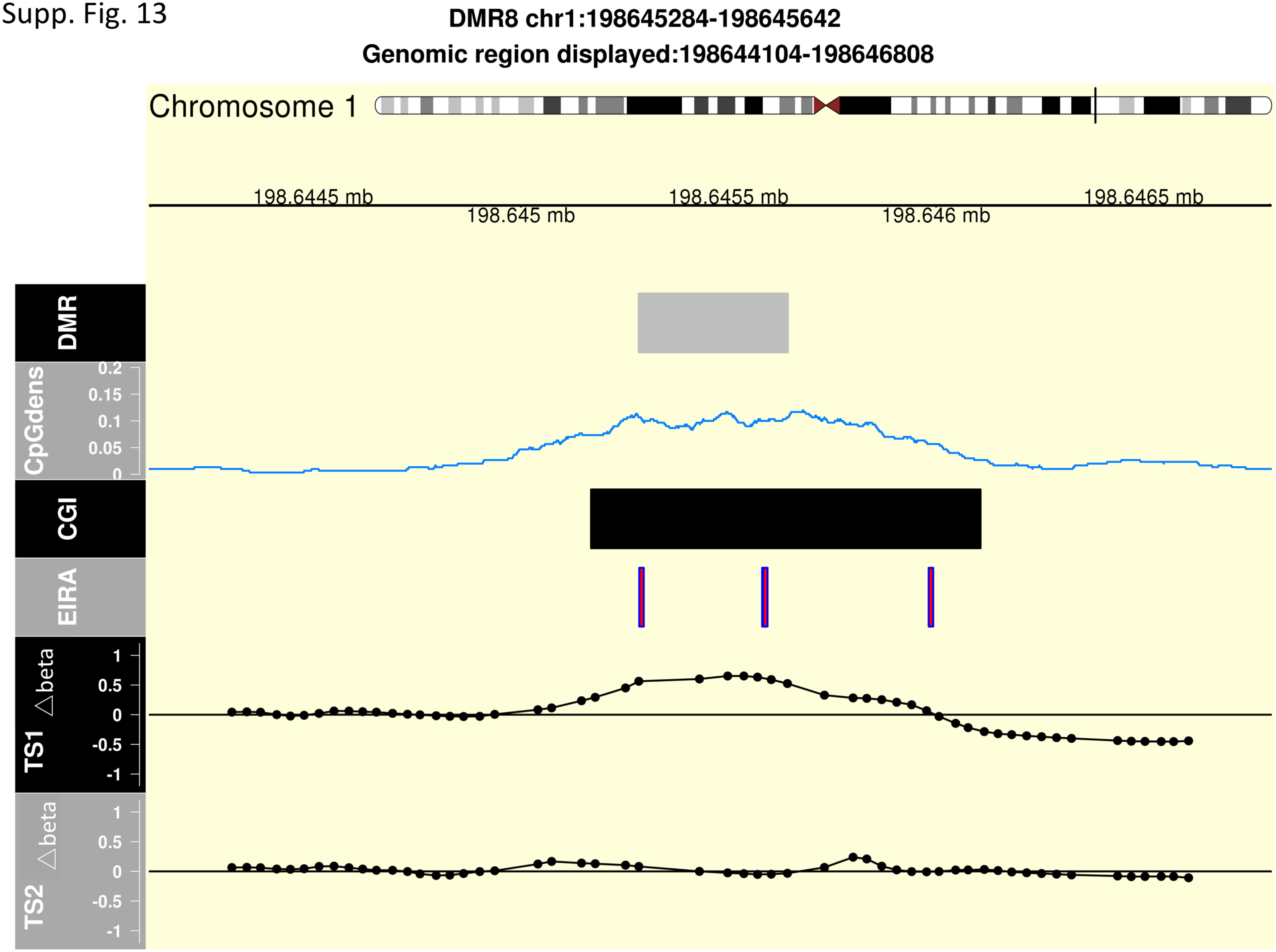

DMR8 chr1:198645284-198645642
Genomic region displayed:198644104-198646808
Chromosome 1
198.6445 mb
198.645 mb
198.6455 mb
198.646 mb
198.6465 mb
DMR
CpGdens
0.2
0.15
0.1
0.05
0
CGI
EIRA
TS1
△beta
1
0.5
0
-0.5
-1
TS2
△beta
1
0.5
0
-0.5
-1

Supp. Fig. 14

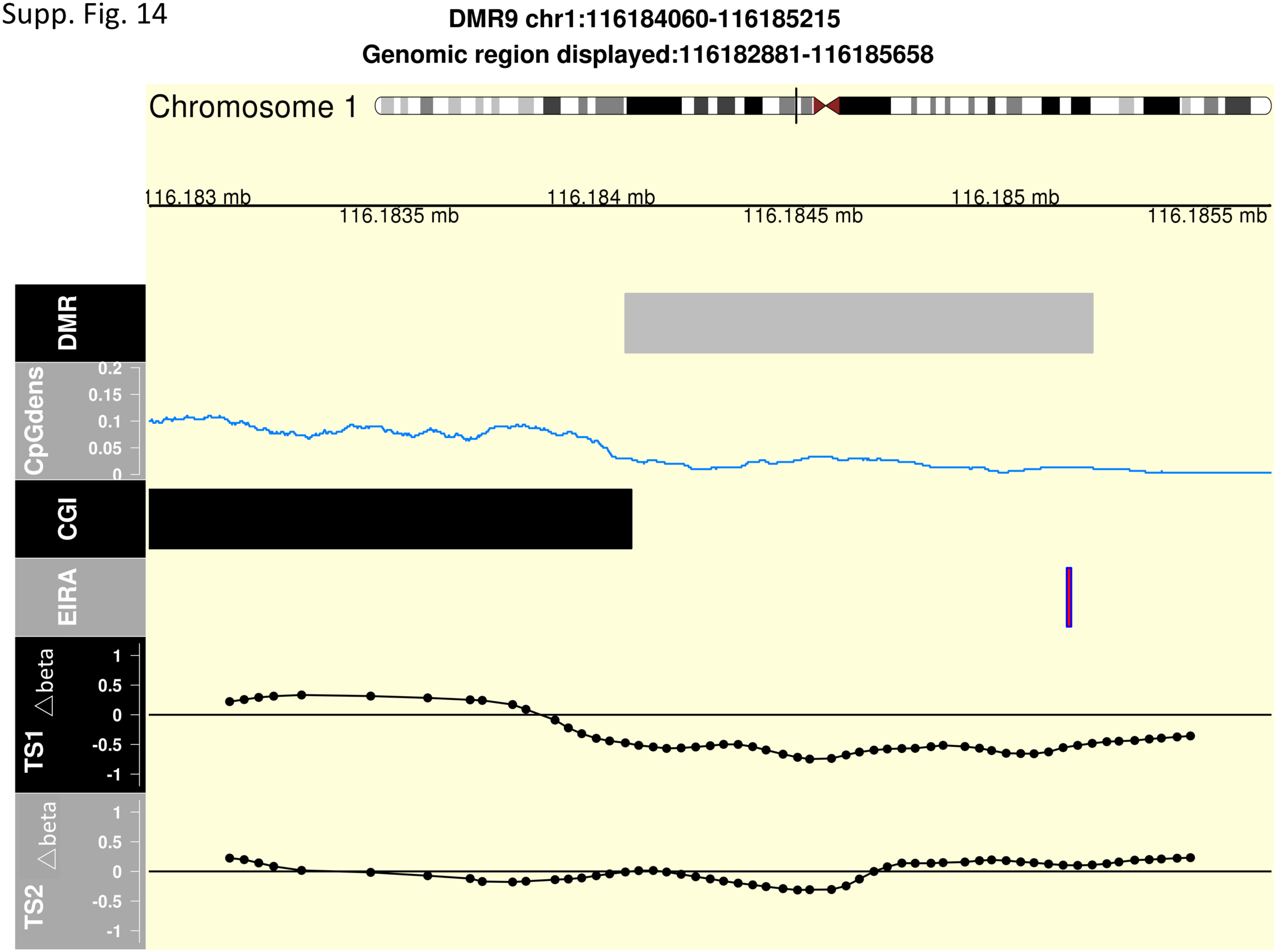
DMR9 chr1:116184060-116185215
Genomic region displayed:116182881-116185658
Chromosome 1
116.183 mb
116.1835 mb
116.184 mb
116.1845 mb
116.185 mb
116.1855 mb
DMR
CpGdens
0.2
0.15
0.1
0.05
0
CGI
EIRA
TS1
△beta
1
0.5
0
-0.5
-1
TS2
△beta
1
0.5
0
-0.5
-1

Supp. Fig. 15

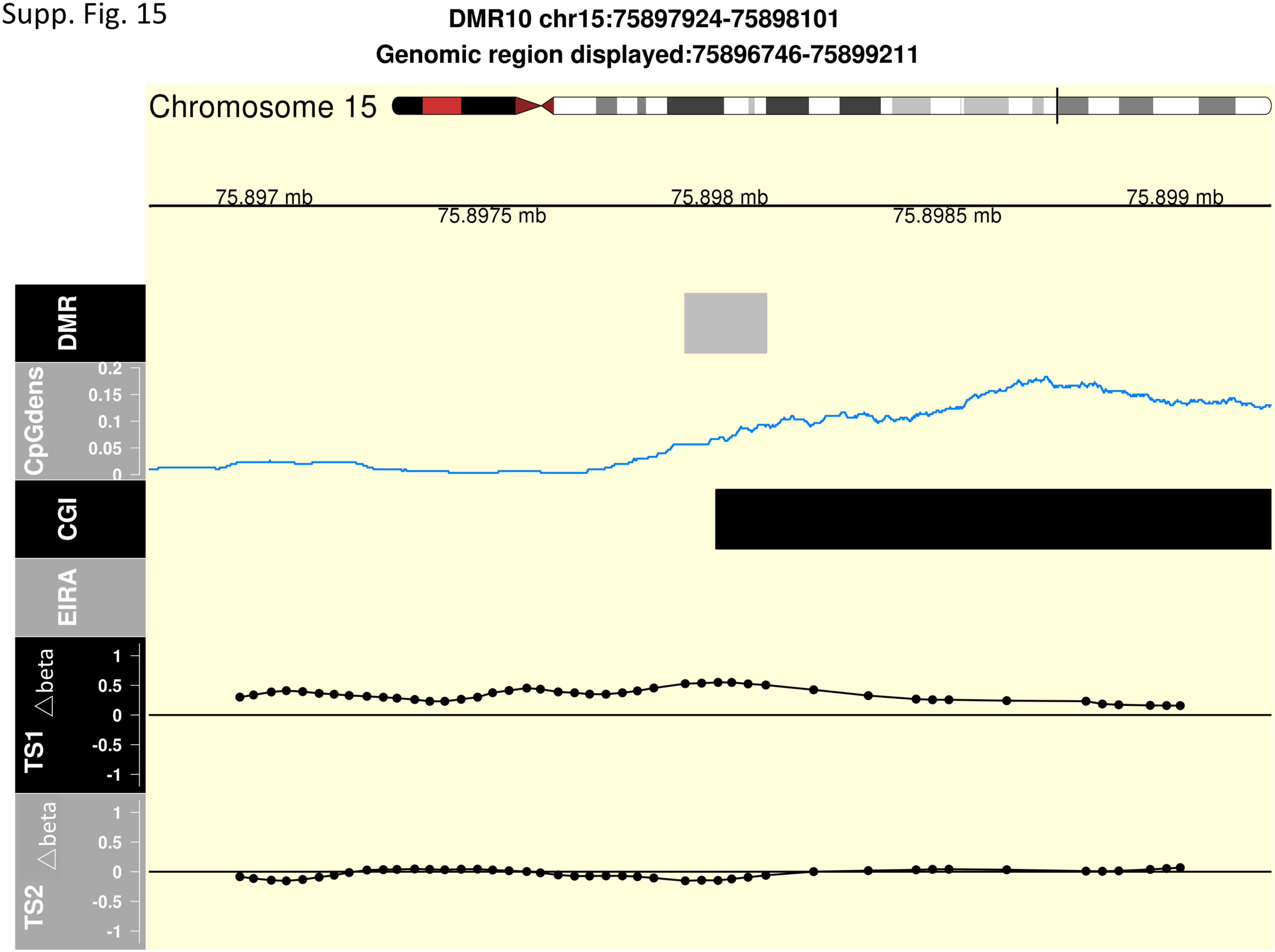

DMR10 chr15:75897924-75898101
Genomic region displayed:75896746-75899211
Chromosome 15
75.897 mb
75.8975 mb
75.898 mb
75.8985 mb
75.899 mb
DMR
CpGdens
0.2
0.15
0.1
0.05
0
CGI
EIRA
TS1
△beta
1
0.5
0
-0.5
-1
TS2
△beta
1
0.5
0
-0.5
-1

Supp. Fig. 16

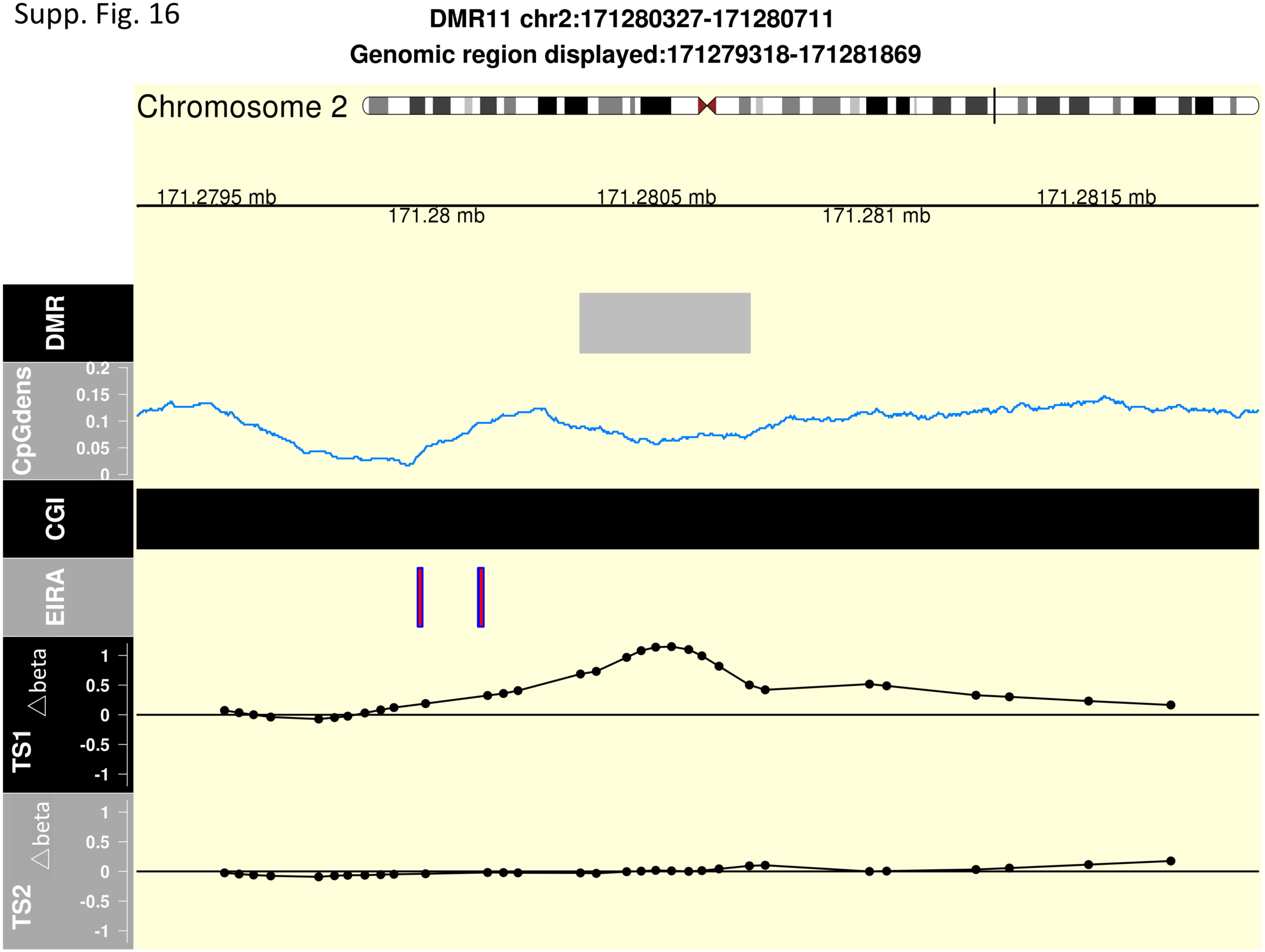

DMR11 chr2:171280327-171280711
Genomic region displayed:171279318-171281869
Chromosome 2
171.2795 mb
171.28 mb
171.2805 mb
171.281 mb
171.2815 mb
DMR
CpGdens
0.2
0.15
0.1
0.05
0
CGI
EIRA
TS1
△beta
1
0.5
0
-0.5
-1
TS2
△beta
1
0.5
0
-0.5
-1

Supp. Fig. 17

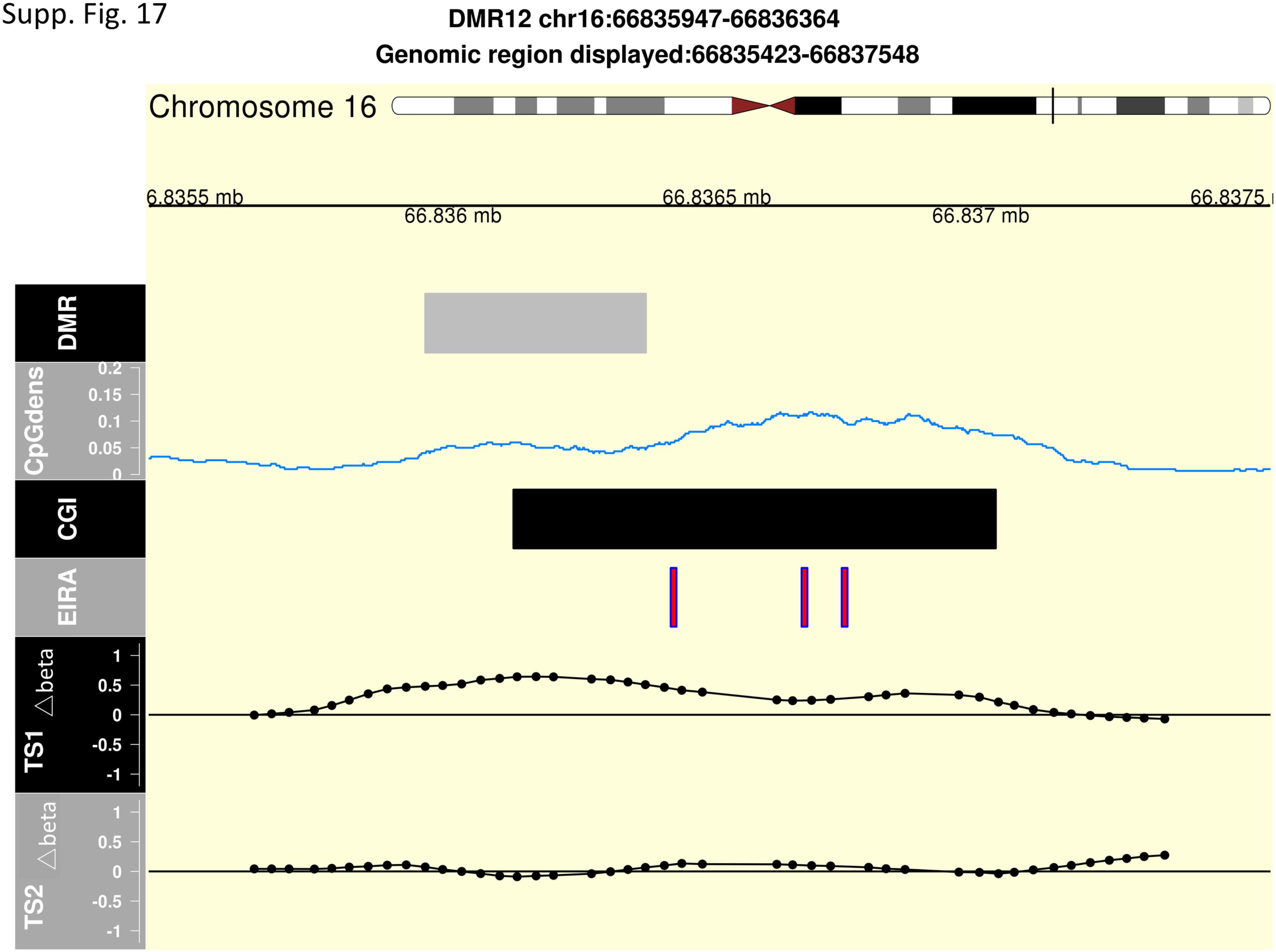

DMR12 chr16:66835947-66836364
Genomic region displayed:66835423-66837548
Chromosome 16
6.8355 mb
66.836 mb
66.8365 mb
66.837 mb
66.8375
DMR
CpGdens
0.2
0.15
0.1
0.05
0
CGI
EIRA
TS1
△beta
1
0.5
0
-0.5
-1
TS2
△beta
1
0.5
0
-0.5
-1

Supp. Fig. 18

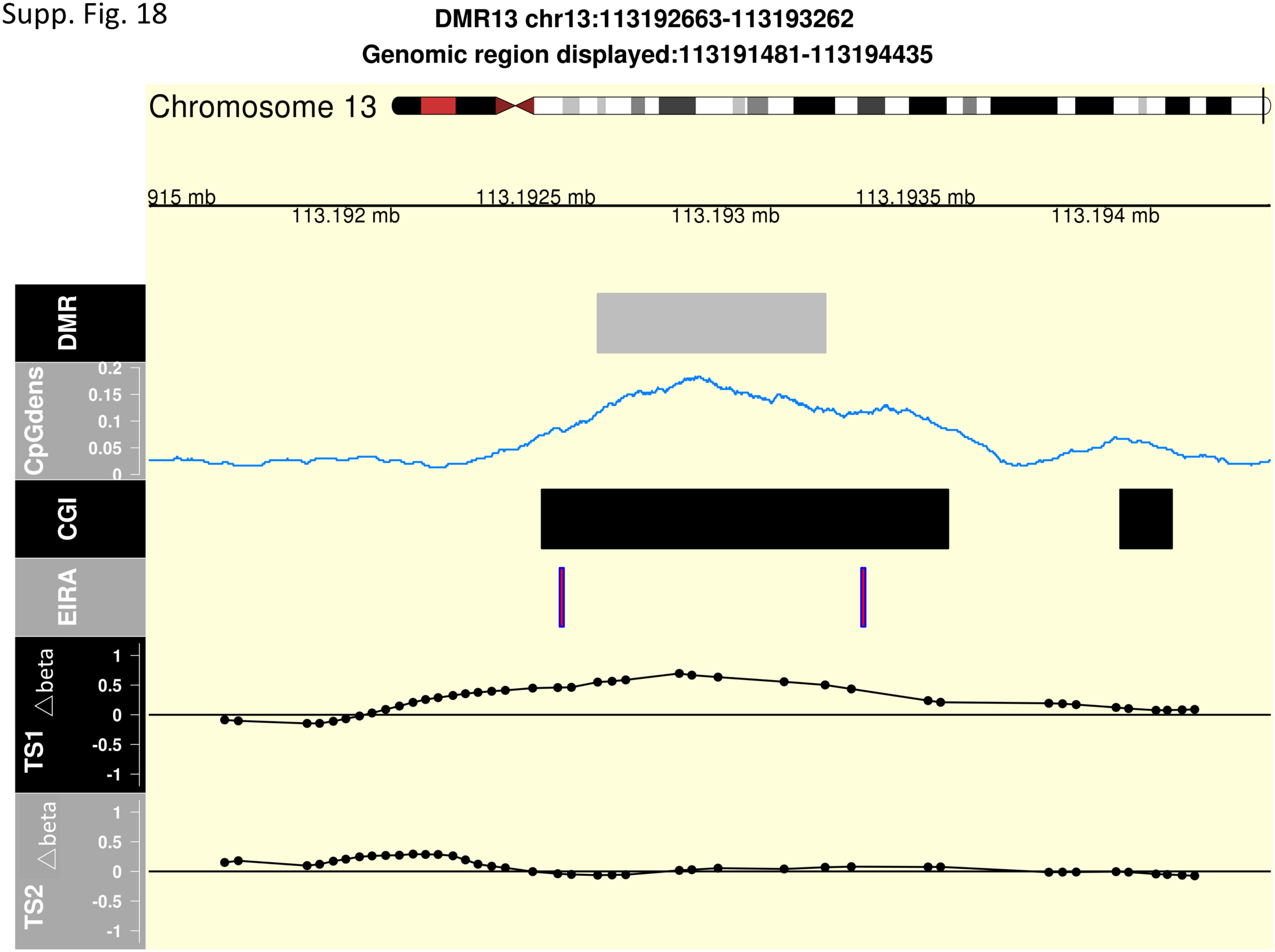
DMR13 chr13:113192663-113193262
Genomic region displayed:113191481-113194435
Chromosome 13
915 mb
113.192 mb
113.1925 mb
113.193 mb
113.1935 mb
113.194 mb
DMR
CpGdens
0.2
0.15
0.1
0.05
0
CGI
EIRA
TS1
△beta
1
0.5
0
-0.5
-1
TS2
△beta
1
0.5
0
-0.5
-1

Supp. Fig. 19

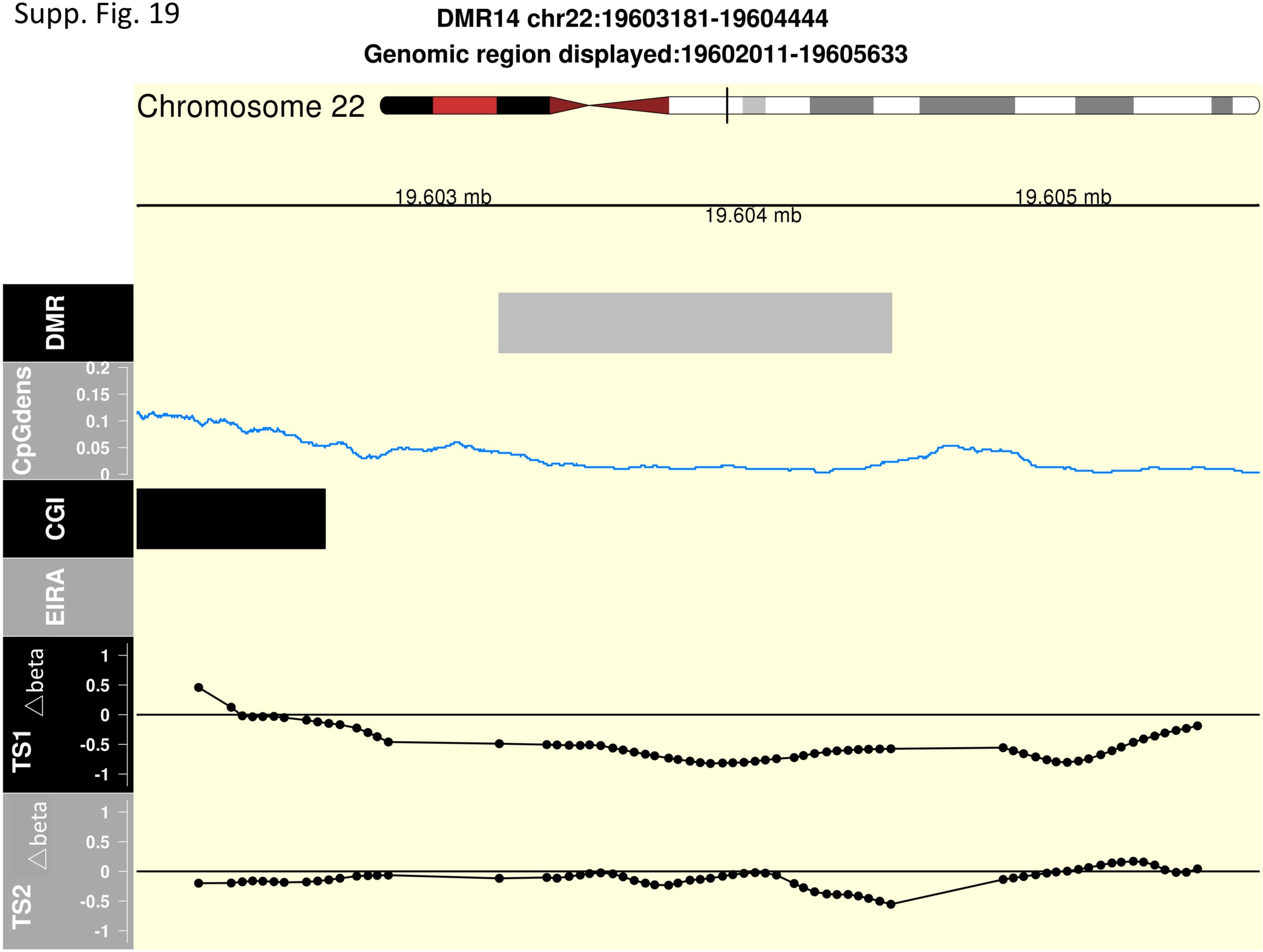
DMR14 chr22:19603181-19604444
Genomic region displayed:19602011-19605633
Chromosome 22
19.603 mb
19.604 mb
19.605 mb
DMR
CpGdens
0.2
0.15
0.1
0.05
0
CGI
EIRA
TS1
△beta
1
0.5
0
-0.5
-1
TS2
△beta
1
0.5
0
-0.5
-1

Supp. Fig. 20

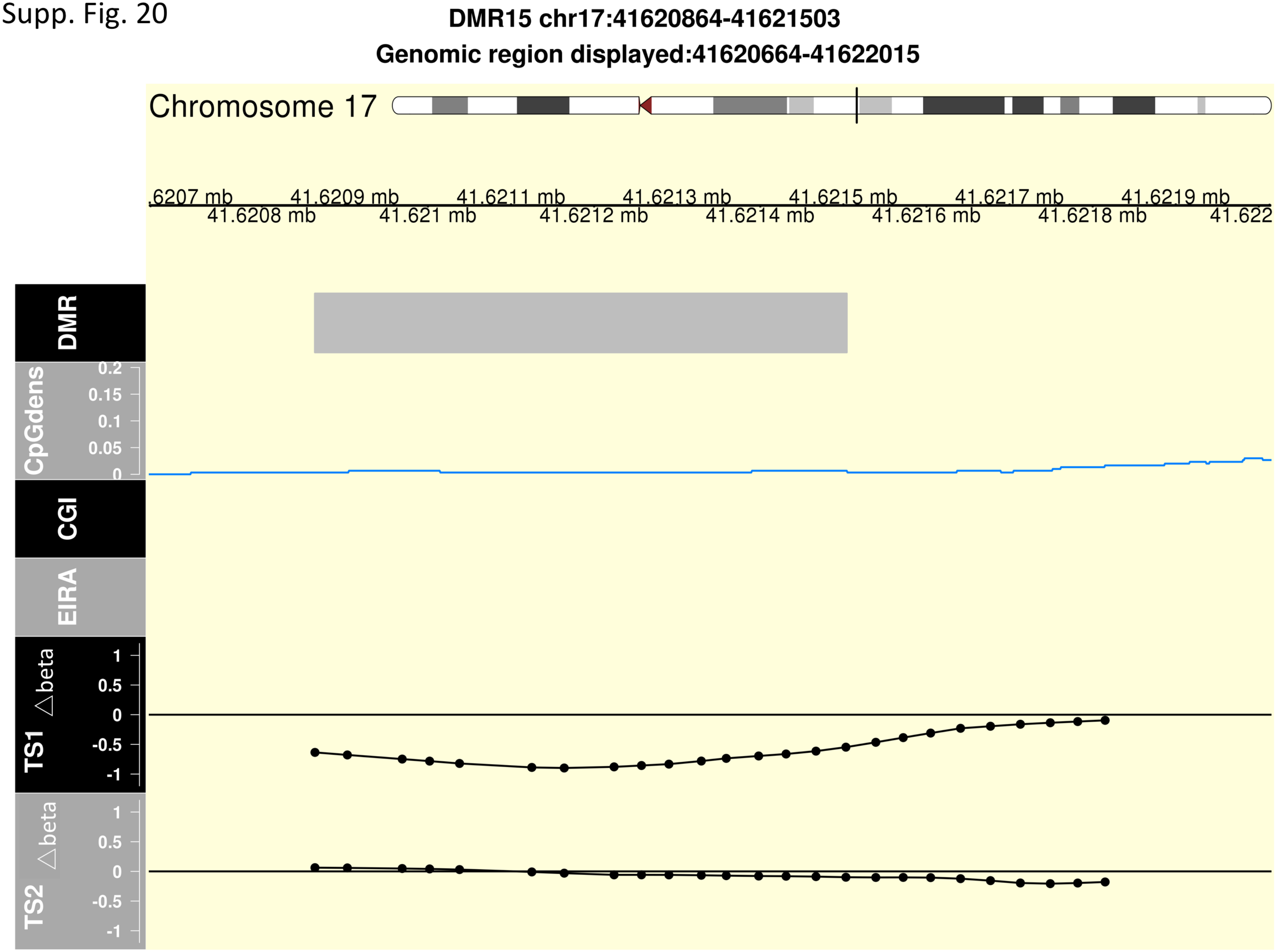

DMR15 chr17:41620864-41621503
Genomic region displayed:41620664-41622015
Chromosome 17
.6207 mb
41.6208 mb
41.6209 mb
41.621 mb
41.6211 mb
41.6212 mb
41.6213 mb
41.6214 mb
41.6215 mb
41.6216 mb
41.6217 mb
41.6218 mb
41.6219 mb
41.622
DMR
CpGdens
0.2
0.15
0.1
0.05
0
CGI
EIRA
TS1
△beta
1
0.5
0
-0.5
-1
TS2
△beta
1
0.5
0
-0.5
-1

Supp. Fig. 21

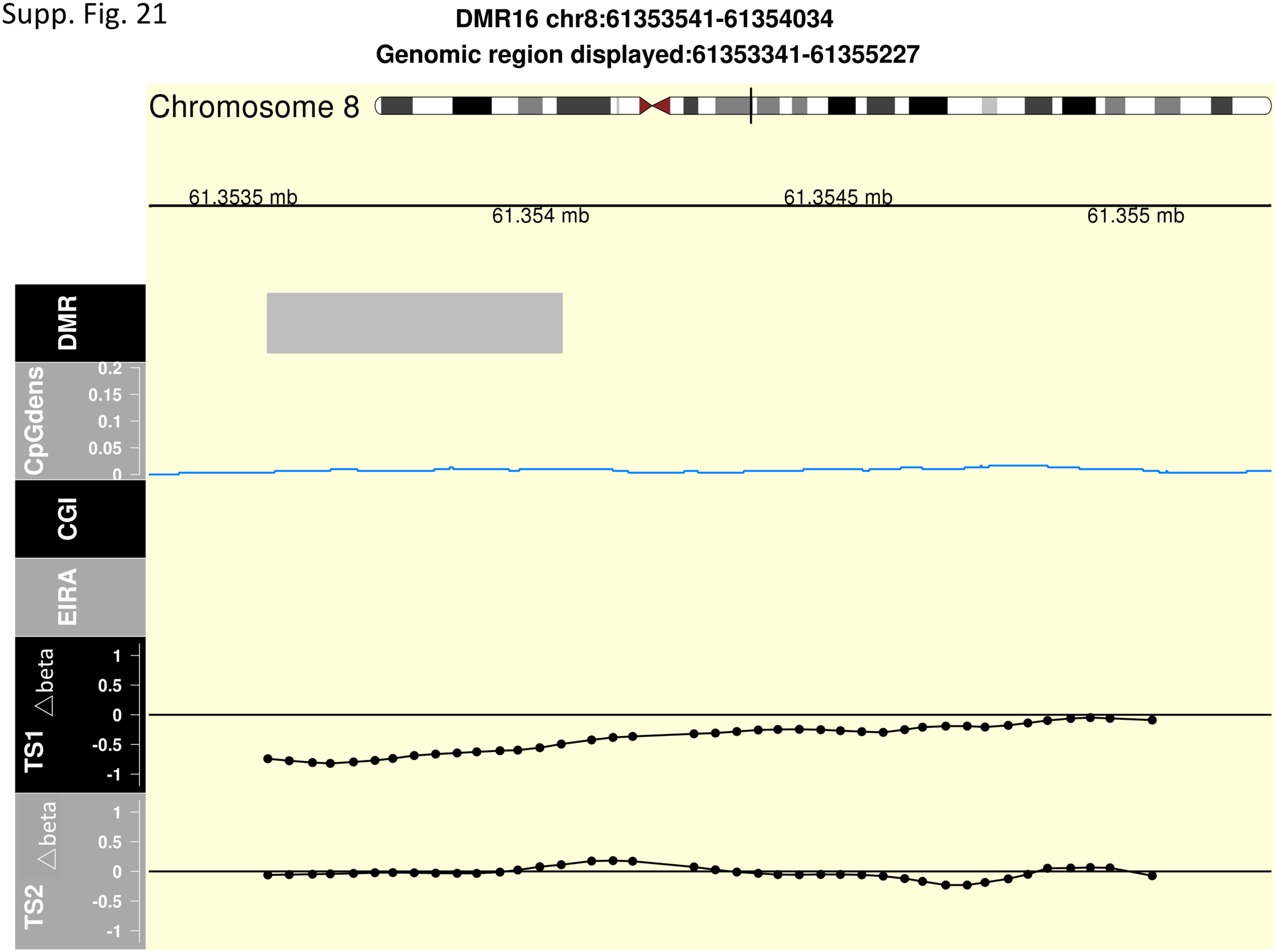
DMR16 chr8:61353541-61354034
Genomic region displayed:61353341-61355227
Chromosome 8
61.3535 mb
61.354 mb
61.3545 mb
61.355 mb
DMR
CpGdens
0.2
0.15
0.1
0.05
0
CGI
EIRA
TS1 △beta
1
0.5
0
-0.5
-1
TS2 △beta
1
0.5
0
-0.5
-1

Supp. Fig. 22

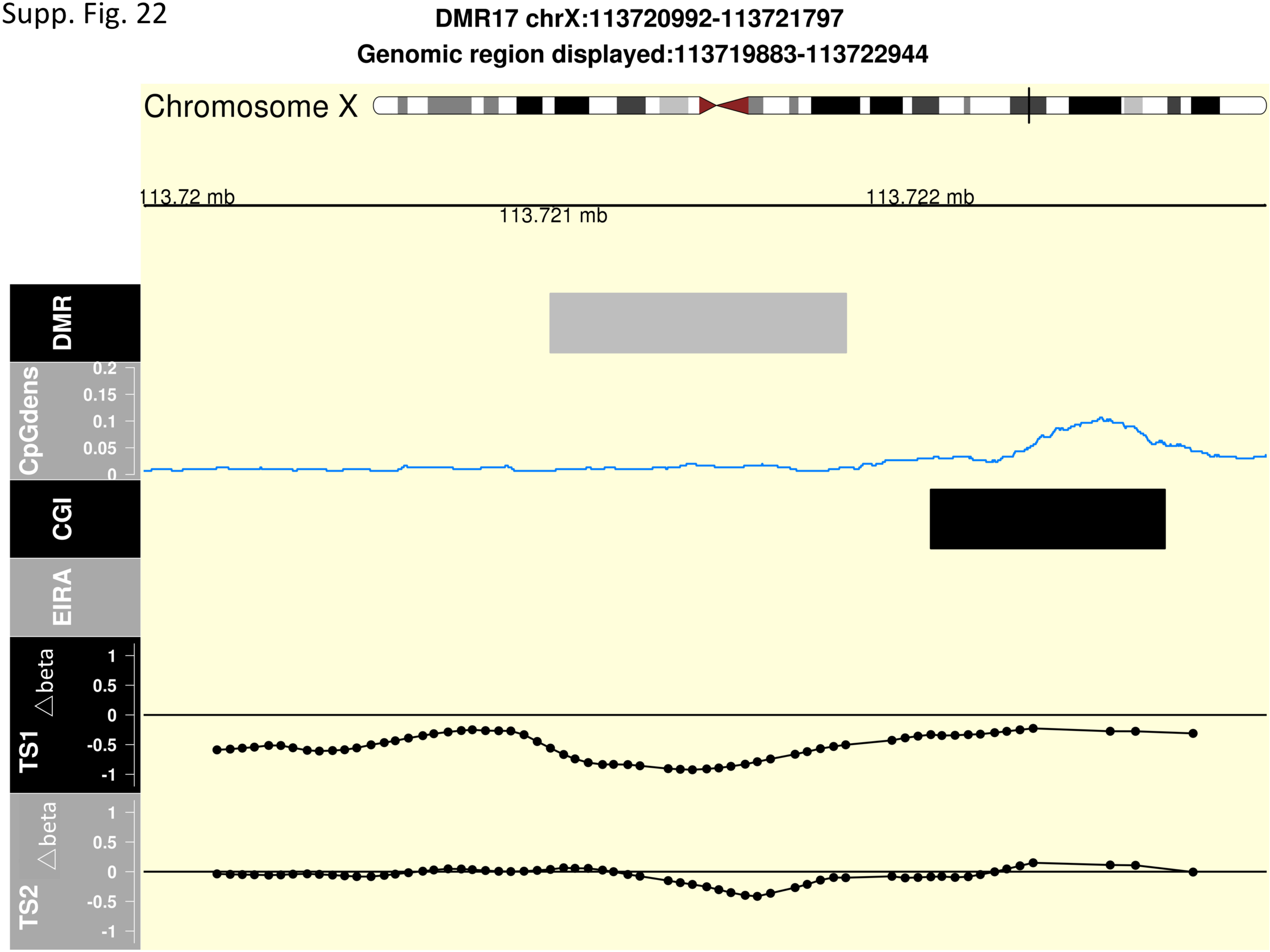

DMR17 chrX:113720992-113721797
Genomic region displayed:113719883-113722944
Chromosome X
113.72 mb
113.721 mb
113.722 mb
DMR
CpGdens
0.2
0.15
0.1
0.05
0
CGI
EIRA
TS1
△beta
1
0.5
0
-0.5
-1
TS2
△beta
1
0.5
0
-0.5
-1

Supp. Fig. 23

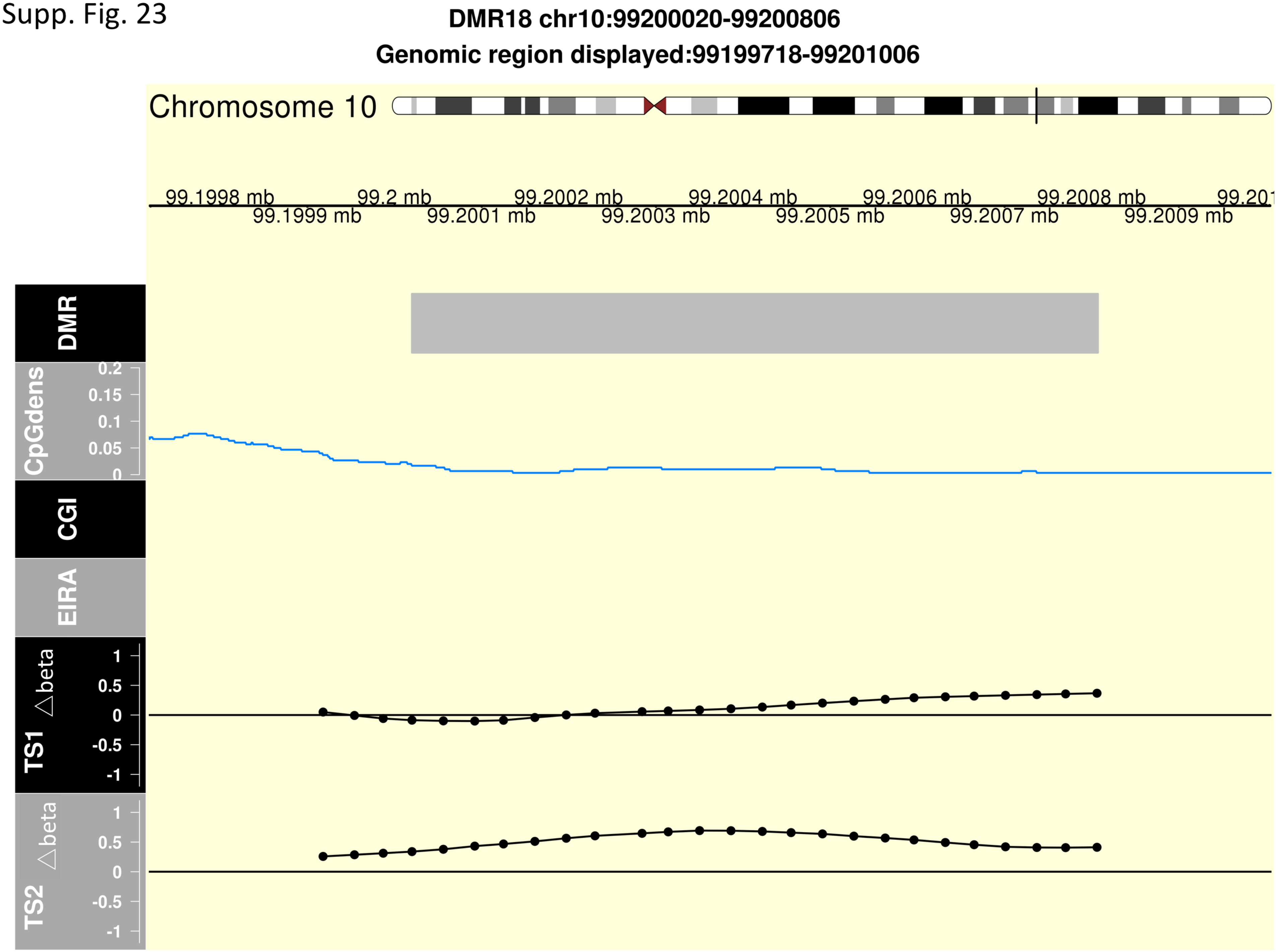
DMR18 chr10:99200020-99200806
Genomic region displayed:99199718-99201006
Chromosome 10
99.1998 mb
99.1999 mb
99.2 mb
99.2001 mb
99.2002 mb
99.2003 mb
99.2004 mb
99.2005 mb
99.2006 mb
99.2007 mb
99.2008 mb
99.2009 mb
DMR
CpGdens
0.2
0.15
0.1
0.05
0
CGI
EIRA
TS1 △beta
1
0.5
0
-0.5
-1
TS2 △beta
1
0.5
0
-0.5
-1

Supp. Fig. 24

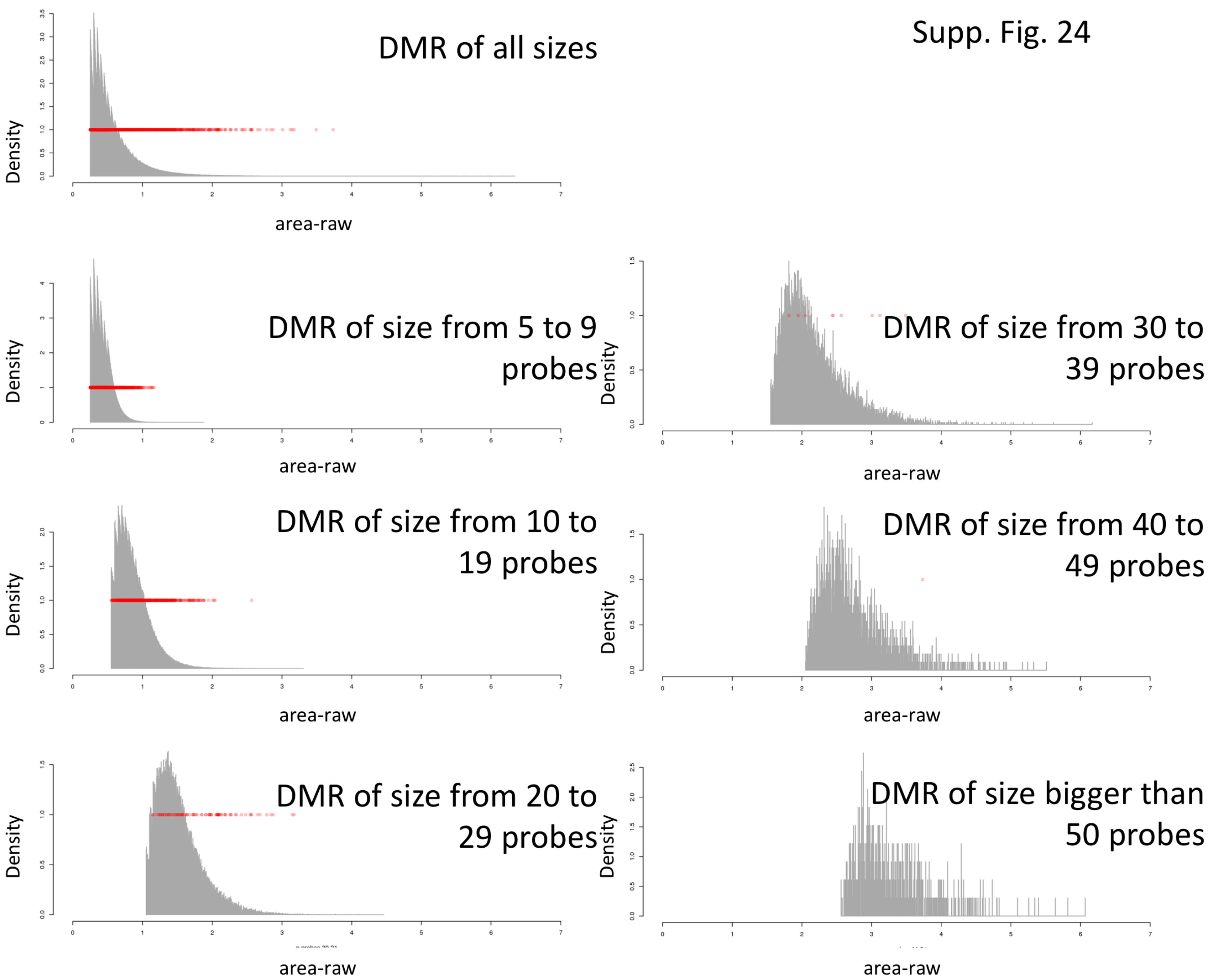
DMR of all sizes
Density
area-raw
DMR of size from 5 to 9 probes
Density
area-raw
DMR of size from 30 to 39 probes
Density
area-raw
DMR of size from 10 to 19 probes
Density
area-raw
DMR of size from 40 to 49 probes
Density
area-raw
DMR of size from 20 to 29 probes
Density
area-raw
DMR of size bigger than 50 probes
Density
area-raw

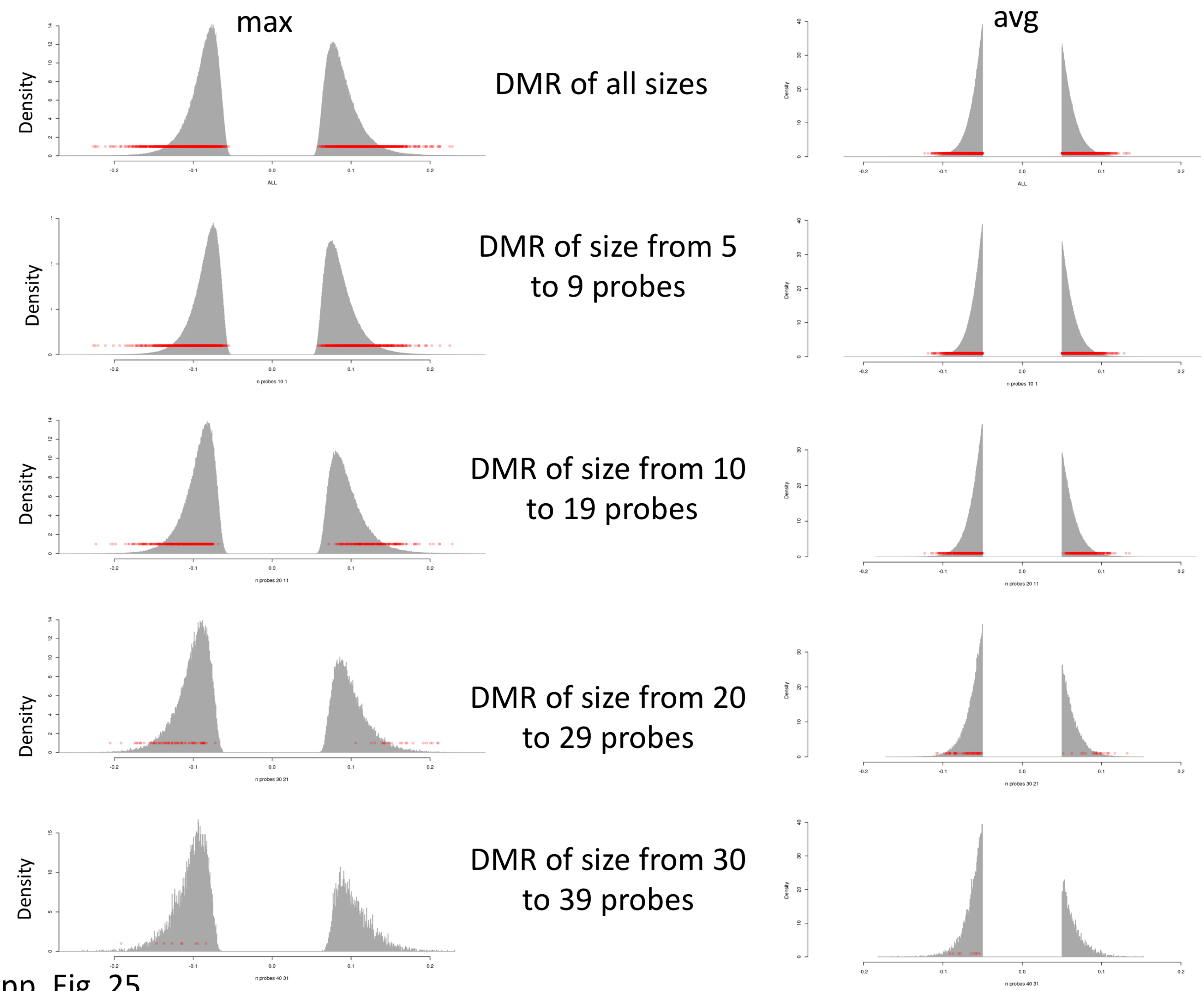
max
avg
DMR of all sizes
DMR of size from 5 to 9 probes
DMR of size from 10 to 19 probes
DMR of size from 20 to 29 probes
DMR of size from 30 to 39 probes
Density
Density
Density
Density
Density
ALL
n probes 10 1
n probes 20 11
n probes 30 21
n probes 40 31
ALL
n probes 10 1
n probes 20 11
n probes 30 21
n probes 40 31

Supp. Fig. 25

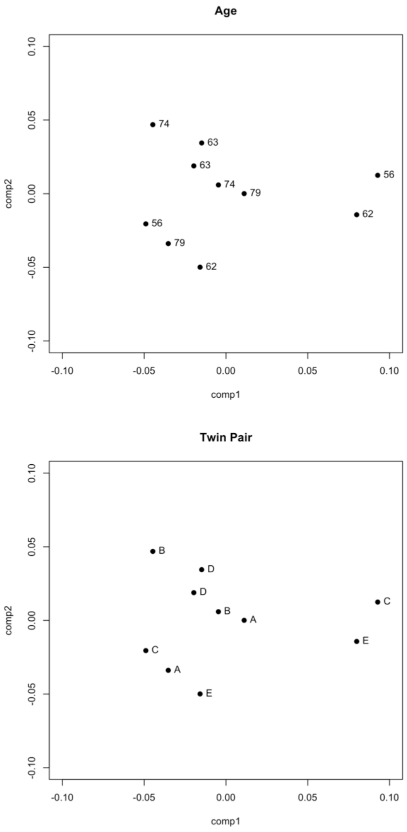


(a) TS1

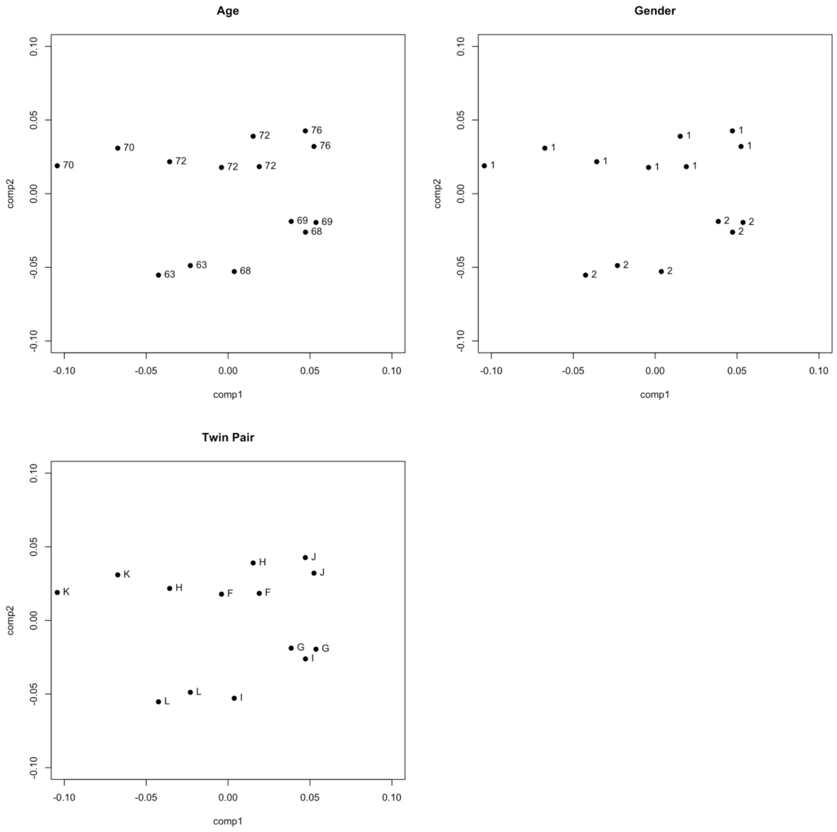


(b) TS2

Supp. Fig. 26

# High-specificity bioinformatics framework for epigenomic profiling of discordant twins reveals specific and shared markers for ACPA and ACPA-positive rheumatoid arthritis

## Additional Figure Legends

**Additional Figure 1. CHARM: distribution of probes.** The plot was generated with ChIPseeker Bioconductor package [1]. The probes are characterized based on their overlapping with gene locations. Promoters are considered as the genomic region covering 1300 upstream and downstream from the Transcription Starting Site.

**Additional Figure 2. Volcano plot. Differential Methylation summary without considering cell proportion correction**. Log-transformed p-value (y-axis) versus log-transformed Fold Change from the associated linear model. A negative (positive) logFC denotes hypomethylation (hypermethylation) in TS1 and TS2 contrasts; the logFC was computed by using M values (see MM) in a linear model. The horizontal and vertical lines are arbitrary thresholds selected to highlight possible tendencies. (a) Provides the results from ACPA positive healthy vs ACPA negative healthy twin siblings (TS1). (b) Provides the results from ACPA positive RA vs ACPA negative healthy twin siblings (TS2).

**Additional Figure 3. PCDHB5 associated DMR from TS2 analysis without cell proportion correction (chr5:140494633-140495503).** Upper panel describes the DMR methylation profile as obtained from the CHARM experiment; the top panel shows the levels of methylation for individual probes for ACPA positive RA twin (red) and ACPA negative healthy twin (blue); lines denote the average methylation profile within each group. Middle panel shows the individual (points) and average (line) differences in methylations between each twin pair. The bottom panel

presents the DMR location. Lower panel CHARM analysis and pyrosequencing data for validation are compared. The two top panels are zooming in over what is shown in top two upper panels; the two bottom panels provide similar information but for pyro-sequencing validation experiments.

**Additional Figure 4. COL13A1 associated TS2 without cell proportion correction DMR (chr10:71229788-71230625).** The upper plot describes the DMR methylation profile as obtained from CHARM experiment. In the upper plot the top panel shows the levels of methylation for individual probes for ACPA positive RA twin (red) and ACPA negative healthy twin (blue); lines denote the average methylation profile within each group. Middle panel shows the individual (points) and average (line) differences in methylations between each twin pair. The bottom panel presents the DMR location. In the bottom plot the CHARM analysis and pyrosequencing data for validation are compared. The two top panels are zooming in over what is shown in top two upper panels; the two bottom panels provide similar information but for pyrosequencing validation experiments.

**Additional Figure 5. SLITRK2 associated TS2 without cell proportion correction DMR (chrX:144705098-144705656)**. The upper plot describes the DMR methylation profile as obtained from CHARM experiment. In the upper plot the top panel shows the levels of methylation for individual probes for ACPA positive RA twin (red) and ACPA negative healthy twin (blue); lines denote the average methylation profile within each group. Middle panel shows the individual (points) and average (line) differences in methylations between each twin pair. The bottom panel presents the DMR location. In the bottom plot the CHARM analysis and pyrosequencing data for validation are compared. The two top panels are zooming in over what is shown in top two upper panels; the two bottom panels provide similar information but for pyrosequencing validation experiments.

**Additional Figure 6. Association of PCDHB5 DMRs (Additional Fig. 3) in TS2 (without cell-proportion correction) to cell-specific methylation markers.** (1) ESTIMATED B VALUE provides the percentage of methylation for every individual (dots) and the average for every clinical group (line). Every vertical group of points denote the position of a probe. (2) DMR: Location of *TS2* DMR obtained without cell proportion correction. (3) CGI: CpG island location (black box).

**Additional Figure 7-23. DMR depicted from DMR2 to DMR18 (See Table 3 and 4).** Description is similar to that in Figure 3. DMR: denotes DMR location (grey box); CpGdens denotes CpG density as computed by CHARM [2] (CpG); CGI: denotes the location of CpG islands (black box); EIRA: location of significantly differentially methylated probes after cell-proportion correction in [3] (blue box with red borders). TS1 △beta (TS2 △beta) shows the smoothed linear slope (differences in methylation or *delta*) associated to ACPA positive healthy (ACPA positive RA twin) in the linear model which is used charm [2] to identify DMR candidates. Every point denotes a probe location.

**Additional Figure 24. Analysis of the statistics used in the dmrFind: area.raw.** The figure shows that the area.raw statistic is dependent of the number of probes in the DMR candidate regions, therefore selecting those with larger number of probes. In each plot the grey area denotes the density function of the boot-strapping-generated DMR-area.raw values; red points denote area.raw values of the candidate DMR regions found in the data.

**Additional Figure 25. Analysis of the statistics used in the dmrFind: max and avg.** The figure shows the max (left plots) and avg (right plots) statistics for randomly generated DMRs (in grey) and for candidate DMR regions (red) for different selection of DMRs based on their number of probes.

**Additional Figure 26. Quality Control: Multi-Dimensional Scaling (MDS).** The figure depicts the MDS plots for TS1 (panel (a)) and TS2 (panel (b)) samples. In each case the same MDS plot is depicted with labels associated to Age (top-left), Gender (top-right) and Twin Pair (bottom-left). None of those variables are identified as significant confounder.

## References


1. Yu, G., Wang, L.G., and He, Q.Y. (2015). ChIPseeker: an R/Bioconductor package for ChIP peak annotation, comparison and visualization. Bioinformatics 31, 2382-2383.
2. Aryee, M.J., Wu, Z., Ladd-Acosta, C., Herb, B., Feinberg, A.P., Yegnasubramanian, S., and Irizarry, R.A. (2011). Accurate genome-scale percentage DNA methylation estimates from microarray data. Biostatistics 12, 197-210.
3. Liu, Y., Aryee, M.J., Padyukov, L., Fallin, M.D., Hesselberg, E., Runarsson, A., Reinius, L., Acevedo, N., Taub, M., Ronninger, M., et al. (2013). Epigenome-wide association data implicate DNA methylation as an intermediary of genetic risk in rheumatoid arthritis. Nat Biotechnol 31, 142-147.
