# Supplementary material for: High-specificity bioinformatics framework for epigenomic profiling of discordant twins reveals specific and shared markers for ACPA and ACPA-positive rheumatoid arthritis
Source: Genome Med. 2016 Nov 22;8:124. doi: 10.1186/s13073-016-0374-0 (PMC5120506; doi:10.1186/s13073-016-0374-0)
Supplement: Additional file 2: — Supplementary Tables S1 to S4. (ZIP 31 kb) [file 13073_2016_374_MOESM2_ESM.zip › 13073_2016_374_MOESM2_ESM.docx]

**Supplemental Table Legends**

**Supp. Table 1. Monozygotic twin pairs discordant for rheumatoid arthritis, age at blood sampling, gender, smoking habits, HLA-se genotype, anti-ccp status and medication at time of blood donation.**

**Supp. Table 2. DMRs identified without cell type correction.** The table shows DMRs (differentially methylated probes) identified after cell type correction in TS1 and TS2; the criteria for inclusion is FWER (family wise error rate) <0.10 in at least one of the three statistics (*italics*). **chr** denotes the chromosome. **Start** (**end**) denotes the start (end) location of the DMR in the chromosome. **nprobes** denotes the number of probes in the DMR. **FWER avg**, **FWER max** and **FWER area** denote the FWER associated to each one of the statistics used (See Materials and Methods); **Permuted p-value** denotes de p-value computed for each DMR locally, comparing each DMR statistics to random permutations of the samples. **TYPE** denotes if the DMR is associated to TS1 (ACPA/healthy discordant) or TS2 (ACPA-positive RA/healthy discordant). The genome build used is hg18.

**Supp. Table 3. DMRs association with clinical variables.** The table shows the outcomes from investigating if the methylation level was associated with Age, Gender, HLA epitope or Smoking in TS1 and TS2 separately for each DMR. Details of the analysis are provided in Materials and Methods: *Changes in DMRs vs confounders*). The values shown are the p-values from linear model analysis; reddish background identifies close-to-significant associations.

**Supp. Table 4. Validation of most relevant DMRs.** Cont. denotes the contrast considered TS1 or TS2. Ratio denotes the percentage of twin pairs that the difference in methylation computed by CHARM and by pyro are in the same direction. Blue marks in Exp. column denotes results identified in CHARM analysis without cell correction (for instance we are not having a significant association for PCDHB14 in TS2 without cell correction); an orange mark denotes results identified in CHARM analysis with cell-correction; and finally, a green mark denotes results identified in the Projection analysis (see Materials and Methods: Projection). P-value and slope denotes the p-values and slopes computed in a linear model analysis (see details in Materials and Methods: *Statistical analysis for validation*). Meta-analysis columns provide the p-values of the meta-analysis conducted using the pyro-sequencing data and combining results from the (unpaired) technical verification and the replication cohort. Each column shows the p-values from two different methodologies: (a) P-value based meta-analysis by “summation of p-value” method and (b) Effect-size based Meta-analysis considering fixed effects (see Materials and Methods: Meta-analysis). In purple are highlighted the significant results (p-values <0.05) from the meta-analysis
