# Supplementary material for: High-specificity bioinformatics framework for epigenomic profiling of discordant twins reveals specific and shared markers for ACPA and ACPA-positive rheumatoid arthritis
Source: Genome Med. 2016 Nov 22;8:124. doi: 10.1186/s13073-016-0374-0 (PMC5120506; doi:10.1186/s13073-016-0374-0)
Supplement: Additional file 3: — Supplementary methods. The file includes extended description of aspects of the bioinformatic analysis. (DOCX 25 kb) [file 13073_2016_374_MOESM3_ESM.docx]

**Additional Methods.**

**High-specificity bioinformatics framework for epigenomic profiling of discordant twins reveals specific and shared markers for ACPA and ACPA-positive rheumatoid arthritis**

**CHARM**: CHARM is a method developed to analyze genome wide gene specific methylation. CHARM methodology combines (1) a tiling array that covers CpGs also in lower CpG density regions of the genome, in addition to CpG islands and shores; a (2) DNA methylation enrichment by fractionation by McrBC and (3) a statistical procedure that involves the identification of consecutive differential methylation sites, identifying them as candidate DMRs after employing a smoothing algorithm allowing the correction for CpG density and fragment biases which may otherwise occur in methyl-enrichment or methyl-depletion DNA fractionation methods. We applied CHARM (employing the hg18 genome build) using DNA from peripheral blood cells (PBC), from 12 monozygotic twin pairs discordant for RA and ACPA to determine the locus specific differential methylation patterns. The experimental part of the method is comprehensively described in[^1^](#_ENREF_1). Briefly, DNA is sheared into sizes ranging from 1500-3000 bases. These fragments are either mock digested or digested with the restriction enzyme McrBC, recognizing Pu^m^C(N_40-3000_)^m^CPu, i.e. cutting any DNA containing methylcytosine on one or both strands. The untreated sample represents input DNA (UT) and the McrBC treated, the methyl-depleted sample (MD). These two samples are size fractionated on a 1% agarose gel, whole genome amplified, Cy3 (UT) respectively Cy5 (UT) labeled and co-hybridized to a custom 2.1 million feature microarray designed as previously described[^1^](#_ENREF_1). A major strength in CHARM methodology is that both array design and statistical methodology are conceptualized together: on one hand for each region there are large number of probes (similar to tiling-arrays); on the other hand a robust statistical methodology was designed to test the significance of observing several consecutive differential methylated probes. The CHARM analysis interrogates approximately 4 million CpGs of the human methylome and the methodology associated was develop to identify differential methylation in regions (DMRs) in contrast to differentially methylated positions (DMPs) used in most other array-based methylation assays (e.g. Illumina 450K arrays,[^2^](#_ENREF_2)^;^ [^3^](#_ENREF_3)). The CHARM statistical analysis first involves the identification of consecutive differential methylation site, identifying them as candidate DMRs and secondly it uses a boot-strapping approach to compute a significance level for each DMR[^1, REFPACKAGE^](#_ENREF_1).

**Array Pre-processing.** In the processing of CHARM[^1^](#_ENREF_1) arrays several quality controls are considered: (1) the signal of background probes (probes that do not match any genomic sequence and are expected to have very low signals) must be, on average, in the lower quantiles of the signal-based-ranking of the untreated channel probes; we considered a minimum threshold of 80% for an array to be included in the analysis; (2) the standard deviation of untreated channel signals must be small (we considered a maximum of 0.5 for an array to be included), considering that probes are arranged randomly; in addition (after spatial and background correction) (3) the difference between the medians of control (CpG-free regions) and non-control probes must be negative; finally probes with a probe quality lower than 80 were discarded (see charm Bioconductor package for details.). In addition, after normalization, a quality control (4) is applied to ensure that high correlation between samples is observed (samples with very low correlation with the rest would have been investigated as outliers).

**DMR candidate identification**: The algorithm is described in detail in [^1^](#_ENREF_1) we briefly describe the two-step procedure as follows: firstly, the set of regions of “consecutive differentially methylated probes” are identified; secondly, a bootstrapping-based scheme is used to create a null-distribution of selected statistics used to compute FWER values for each region. For the first step, we used the dmrFind function with the default parameters except that we set min.probes to five (minimum number of probes in a DMR) and we used several statistics to define DMRs. Several normalization and pre-processing methodologies were compared (e.g. quantile normalization and increasing the number of permutations) but the selection of DMRs was minimally affected.

**Bootstrapping for statistical validation**: The algorithm also provides three different statistics that are computed for each candidate DMR: a) *avg*, average (across probes) percentage methylation difference, b) *max*, maximum percentage methylation difference, and c) *area.raw*, number of probes multiplied by the averaged difference of methylation, the latter of which is the default mode. By running the default mode we observed that from the list of candidate DMRs, the selection was biased towards DMRs with larger number of probes. To investigate this, we first stored the DMRs computed from 5000 bootstrapping simulations, then we plotted the relation between the statistics and the number of probes in a DMR. Suppl. Fig. 23 shows that using the default statistic (area.raw) it is not possible to compare candidate DMRs with different number of probes as the selection is biased towards candidate DMRs with larger number of probes; this is not true for the other two statistics (see Suppl. Fig. 24). To make a robust statistical validation of candidate DMRs we computed a modified version of FWER computation from CHARM that computes the FWER for each statistics using only resampled-based-DMRs of the same number of probes. To select a final set of statistically-validated DMRs we considered only DMR with at least six probes to reduce the number of false positives (following results from Supp. Fig. 23 and 24). Importantly during each boot-strapping iteration we only selected cDMR that were significant also in a permutation test in order to discard outlier-driven results (see Materials and Methods: Permuted p-value during bootstrapping)

**References**

1. Irizarry, R.A., Ladd-Acosta, C., Carvalho, B., Wu, H., Brandenburg, S.A., Jeddeloh, J.A., Wen, B., and Feinberg, A.P. (2008). Comprehensive high-throughput arrays for relative methylation (CHARM). Genome Res 18, 780-790.

2. Sandoval, J., Heyn, H., Moran, S., Serra-Musach, J., Pujana, M.A., Bibikova, M., and Esteller, M. (2011). Validation of a DNA methylation microarray for 450,000 CpG sites in the human genome. Epigenetics : official journal of the DNA Methylation Society 6, 692-702.

3. Bibikova, M., Barnes, B., Tsan, C., Ho, V., Klotzle, B., Le, J.M., Delano, D., Zhang, L., Schroth, G.P., Gunderson, K.L., et al. (2011). High density DNA methylation array with single CpG site resolution. Genomics 98, 288-295.
